# Indian Red Jungle fowl reveals a genetic relationship with South East Asian Red Jungle fowl and Indian native chicken breeds as evidenced through whole mitochondrial genome sequences

**DOI:** 10.3389/fgene.2023.1083976

**Published:** 2023-08-09

**Authors:** M. Kanakachari, R. N. Chatterjee, M. R. Reddy, M. Dange, T. K. Bhattacharya

**Affiliations:** ^**1**^ ICAR-Directorate of Poultry Research, Hyderabad, India; ^2^ EVA.4 Unit, Faculty of Forestry and Wood Sciences, Czech University of Life Sciences Prague, Prague, Czechia

**Keywords:** chicken, mitochondrial DNA, next-generation sequencing, SNPs, mutations and variants, molecular phylogeny

## Abstract

**Background:** Native chickens are dispersed in a wide geographical range and have hereditary assets that are kept by farmers for various purposes. Mitochondrial DNA (mtDNA) is a widely utilized marker in molecular studies because of its quick advancement, matrilineal legacy, and simple molecular structure.

**Method and Results:** We performed NGS sequencing to investigate mitochondrial genomes and to evaluate the hereditary connections, diversity, and measure of gene stream estimation in Indian native chicken breeds and Red Jungle fowl. The chicken breeds were genotyped using the *D-loop* region and 23 haplotypes were identified. When compared to Indian native breeds, more haplotypes were identified in the NADH dehydrogenase subunits, *Cytochrome c oxidase*, *Cytochrome b*, *ATP synthase subunit 6*, and *Ribosomal RNA genes*. The phylogenetic examination indicated that the analyzed chicken breeds were divided into six significant clades, namely A, B, C, D, E, and F, of which the F clade indicated the domestication of chicken breeds in India. Additionally, our work affirmed that the Indian Red Jungle Fowl is the origin for both reference Red Jungle Fowl as well as all Indian breeds, which is reflected in the dendrogram as well as network analysis based on the whole mtDNA and *D-loop* region. Indian Red Jungle Fowl is distributed as an outgroup, suggesting that this ancestry was reciprocally monophyletic.

**Conclusion:** The mtDNA sequences of Indian native chickens provided novel insights into adaptation mechanisms and the significance of important mtDNA variations in understanding the maternal lineages of native birds.

## Introduction

Modern-day chicken breeds mostly evolved from Red Jungle fowl (RJF), which is evident from archeological discoveries (Darwin, 1868; Beebe, 1918; Danforth, 1958; Morejohn, 1968; Fumihito et al., 1994). There are also some reports on the contribution of other Jungle fowl in evolving many breeds across the Globe (Eriksson et al., 2008; Lawal, 2017). However, according to the available data, it is unclear when and where the first domestication of chickens took place (Zeuner, 1963; Crawford, 1984; West and Zhou, 1988; Fumihito et al., 1996; Liu et al., 2006; Xiang et al., 2014; Peters et al., 2015). In early 3,200 BC, chicken domestication was observed in the Indus valley, and accepted as the epicenter of chicken domestication (Zeuner, 1963). Wild RJF can be found in the forests of South East Asia and India; when people domesticated the chicken and spread it to different parts of the world, the genome landscape of domestic chickens was molded by natural and artificial selection, bringing about a wide range of breeds and ecotypes (Liu et al., 2006; Stevens, 1991; Peterson and Brisbin, 1999; Silva et al., 2009; Gifford-Gonzalez and Hanotte, 2011; Mwacharo et al., 2011). The domestic chicken has broad phenotypic variations. However, the RJF lack the phenotypic variations that would be the result of domestication, for example, plumage color and other morphological characteristics, behavioral and production traits, adaptation to different agro-ecosystems, and rigid human selection for production as well as aesthetic qualities (Schütz et al*.,* 2001; Keeling et al., 2004; Tixier-Boichard et al., 2011). Following domestication, large-scale breeding programs have resulted in more than sixty chicken breeds representing four particular genealogies: egg-type, game, meat-type, and bantamweight (Moiseyeva et al., 2003). According to the taxonomy, the genus *Gallus* is composed of four species: *G. gallus* (Red Jungle Fowl), *G. lafayettei* (Lafayette’s Jungle Fowl), *G. varius* (Green Jungle Fowl), and *G. sonneratii* (Grey Jungle Fowl). At present, RJF has five sub-species based on phenotypic traits and geographic distribution of the populations: *G. g. gallus* (South East Asia RJF), *G. g. spadiceus*, *G. g. bankiva*, *G. g. murghi* (Indian RJF) and *G. g. jabouillei* (Niu et al., 2002). The domestic chicken is considered either a subspecies of RJF (*G. g. domesticus*) or a separate species, *G. domesticus*. Scientists are concerned about the genetic integrity and conservation status of the wild RJF and those held in avicultural assortments. It was revealed that the domestic chicken is hybridized with the wild RJF, resulting in the erosion of the genetic purity of the wild birds (Peterson and Brisbin, 1999; Brisbin et al., 2002; Nishibori et al., 2005). Because the previous examinations depend on phenotypic characters, small samples were utilized for DNA investigations. Mitochondrial *D-loop* sequence phylogeny and nuclear gene analyses demonstrated conceivable hybridization between GJF-RJF/domestic birds (Nishibori et al., 2005). Based on these reports, the evaluation of the genetic uniqueness of Indian RJFs is significant for conservation and population studies.

In the study of chicken hereditary diversity, microsatellites have been effectively utilized (Karsli and Balcıoğlu, 2019). For examining the hereditary connections between chicken populations, hereditary assorted diversity estimates utilizing the exceptionally polymorphic variable number of tandem repeat loci have yielded reliable and precise data. Over the previous decade, the utilization of maternally inherited mitochondrial DNA (mtDNA), particularly its complete displacement-loop (*D-loop*) region, has expanded. To track genetic information about chicken ancestral breeds, demonstrating the phylogenetic relationship, genetic distance, and variability within and between populations, one of the most significant and remarkable molecular tools, the nucleotide sequence of the mitochondrial *D-loop* region, is used (Moore, 1995; Nishibori, 2004). The mtDNA or an explicit part of mtDNA (e.g. *D-loop*) sequencing gives precise data on evolution and hereditary diversity (Fumihito et al., 1994; Di Lorenzo et al., 2015). The *D-loop* region evolves much faster than different areas of the mtDNA and does not encode a protein. A variation study was done utilizing the mtDNA *D-loop* region and *HVI* domain of 397 bp fragments for 398 African native chickens from 12 countries; 12.59% polymorphic sites were discovered (Mobegi, 2006). Likewise, 25 individuals from six native Chinese chicken populations recorded polymorphic variation rates between the range of 7.05% and 5.54%, respectively (Niu et al., 2002; Liu et al., 2004). However, there are no reports on Indian native chickens at the mitochondrial genomic level. Examining native chickens at the mitochondrial genomic level can explain the mitochondrial genomic premise of their disparities and the particular attributes of indigenous chickens can be accurately investigated. The comprehension of phylogeography will clarify the demographic history, origin, and extension of chicken species. To defeat the issue of parallel mutations and lineage exchange between different populaces, network analysis has enhanced phylogenetic trees (Bandelt et al., 1999). India is a huge nation that contains a unique scope of altitudes and climates, meaning native chickens have an astounding genetic diversity. As indicated by ICAR-NBAGR, India has 19 indigenous breeds: *Ankaleshwar*, *Aseel*, *Busra*, *Chittagong* (*Malay*), *Danki*, *Daothigir*, *Ghagus*, *Harringhata Black*, *Kadaknath*, *Kalasthi*, *Kashmir Favorolla*, *Miri*, *Nicobari*, *Punjab Brown*, *Tellichery*, *Mewari*, *Kaunayen chicken*, *Hansli*, and *Uttara* (Mogilicherla et al., 2022). There is a need to characterize native chicken lines at the molecular level to enact protection and improvement activities to benefit the nation. With the extended focus on genetic preservation, remarkable alleles may be helpful when making choices to keep up with native varieties. Therefore, the current examination aims to assess the hereditary divergence between twenty-two native Asian breeds and seven native Indian chicken breeds (i.e. *Aseel*, *Ghagus*, *Nicobari Brown*, *Tellicherry*, *Kadaknath*, *Haringhata Black*, and *Red Jungle Fowl*) utilizing mtDNA NGS sequence data.

## Materials and methods

### Experimental birds and sample collection

**FIGURE 1:**
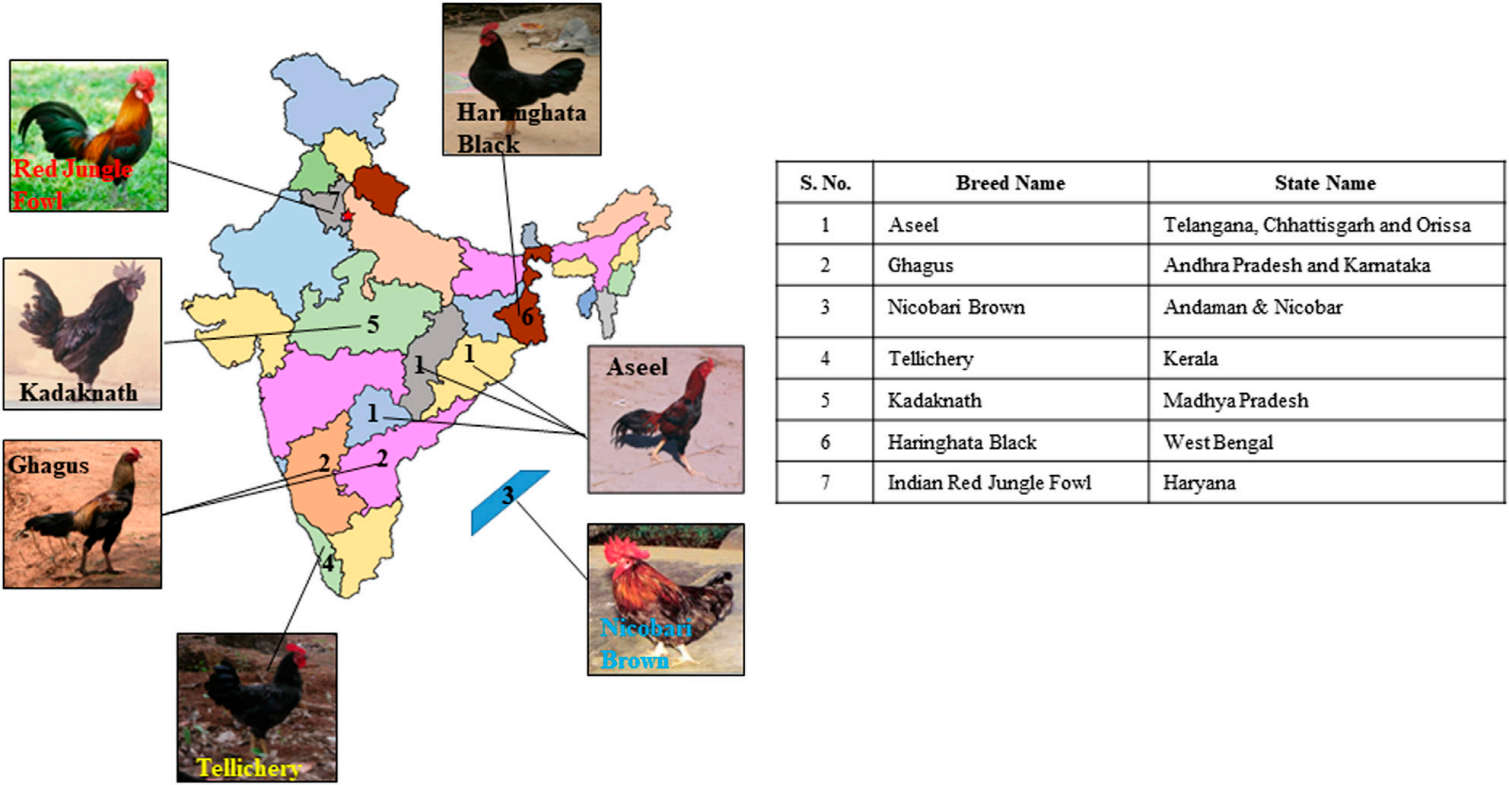
Geographic distribution of native Indian chicken breeds used in the current study.

The seven Indian native chicken breeds, namely *Aseel*, *Ghagus*, *Nicobari brown*, *Kadaknath*, *Tellicherry*, *Haringhata black*, and *Red Jungle fowl*, were studied. Blood samples of five female birds each of *Aseel*, *Ghagus*, *Nicobari brown*, *Nicobari black*, and *Kadaknath* were collected from the experimental farms of Directorate of Poultry Research, Hyderabad while samples of *H. black* and *Red Jungle fowl* were collected from the experimental farms of WBUAFS, West Bengal and CSKHPKVV, Palampur, respectively. The blood samples of *Tellicherry* were collected from the local farmers of Kerala state (Figure 1; Tables 1, 2). The blood samples of each breed (five individual birds) were pooled and stored at −80°C. The DNA was extracted from pooled blood samples according to the lab-standard phenol-chloroform extraction method (Sambrook and Russell, 2001). The experiment was approved by the Institute Animal Ethics Advisory Committee (IAEC) ICAR-Directorate of Poultry Research, Hyderabad, India.

**TABLE 1:** Information on the native Indian chicken breeds used in the current study.

Poultry Breeds and Codes List						
S.No.	Livestock Species	Species Code	Breed Name	Breed Code	Home tract	Accession number
	Fowl	16				
1	Indigenous		Aseel	F010	Chhattisgarh, Orissa and Andhra Pradesh	INDIA_CHICKEN_2615_ASEEL_12002
2			Ghagus	F070	Andhra Pradesh and Karnataka	INDIA_CHICKEN_0108_GHAGUS_12007
3			Harringhata Black	F080	West Bengal	INDIA_CHICKEN_2100_HARRINGHATABLA CK_12008
4			Kadaknath	F090	Madhya Pradesh	INDIA_CHICKEN_1000_KADAKNATH_1200 9
5			Nicobari Brown	F140	Andaman & Nicobar	INDIA_CHICKEN_3300_NICOBARI_12013
7			Tellichery	F160	Kerala	INDIA_CHICKEN_0900_TELLICHERY_12015
8			Indian Red Jungle Fowl	—	—	—

**TABLE 2:** Characteristic features of native Indian chicken breeds used in the current study.

S.No.	Breed Name	Weight (Avg Kg)		Plumage Type	Plumage Pattern	Plumage Colour	Comb Type	Skin Colour	Shank Colour	Egg Shell Colour	Visible Character	Main use
		Male	Female									
1	Aseel	4	2.59	Normal	Patchy	Red, Black	Pea	Yellow	Yellow	Brown	Small but firmly set comb. Bright red ear lobes. Long and slender face devoid of feathers. The general feathering is close, scanty and almost absent on the brest. The plumage has practically no fluff and the feathers are tough.	Socio-Cultural-Game/Fighting; Food-meat
2	Ghagus	2.16	1.43	Normal	Patchy	Brown	Pea or single	White	Yellow	Light Brown	Cocks have shinning bluish black feathers on breast, tail and thighs. Neck is covered with golden feathers. Throat in some cases is loose and hanging. Wattles are small and red in colour. Ear lobes are mostly red.	Food - meat, eggs
3	Harringhata Black	1.284	1.121	Normal	Self-Black	Black	Single	White	Grey	Light Brown	—	Food-Meat, eggs
4	Kadaknath	1.6	1.125	Normal	—	Ranges from silver to gold spangled to blue balck	—	Dark grey	Grey	Light Brown	The colour of day old chicks is bluish to black with irregular dark stripes over the back. In the adults, comb, wattles and tongue are purple. The shining blue tinge of the ear lobes adds to its unique features.	Food - meat; Socio-cultural - religious ceremonies
5	Nicobari Brown	1.8	1.3	Normal	Solid	Black	Single	Dark grey	Grey	Light Brown	The birds are short legged. Shank length at 10 weeks of age varies from 3.50 to 3.85 cm.	Food - Eggs and Mea
6	Tellichery	1.62	1.24	Normal	Solid	Black with shining bluish tinge	Single	Grey	Blackish grey	Light Brown	Shining bluish tinge on hackle, back and tail. Comb is red and large in size. It is erect in cocks and drooping on the rear side in hens. Wattles are red in colour. Ear lobe is mostly red in colour. Eye ring is blackish red. Beak is blackish.	Food - meat, eggs
7	Indian Red Jungle Fowl	2.04	1.36	Normal	Solid	Bright orange and black	Single	White	Yellow	White	Feathered shank, bunch of feather on head (crown structure) in about 18 % birds	Egg, meat and socio-cultural

### Primer designing

Primer designing, library preparation, and sequencing were performed at Genotypic Technology’s Genomics facility. Eight primer pairs were designed to cover the entire mitochondrial genome. About 10 ng of DNA was taken for PCR to amplify explicit products 2–3 kb in size. All the products (five individual birds’ pooled DNA PCR samples) were checked using a 1% Agarose gel (Supplementary Figure S1). All products were pooled in equal amounts for sonication utilizing Covaris S220.

### DNA template library preparation and sequencing by Ion PGM™ sequencer

Library Preparation was done following the Ion Torrent protocol outlined in the Ion plus fragment library kit (ThermoFisher Scientific, United States, # 4471252). Approximately 500 ng of fragmented and cleaned DNA was taken for library preparation. End-repair and adapter ligation was done and the samples were barcoded in this progression. The samples were cleaned utilizing AMPure XP beads. Samples were size-chosen utilizing a 2% low melting agarose gel. The gel-purified samples were amplified for the enrichment of adapter-ligated fragments as per the protocol. The amplified products were cleaned using AMPure XP beads and quantified using Qubit Fluorometer and then run on Bioanalyzer High sensitivity DNA Assay to assess the quality of the library. The purified libraries were then used to prepare clonally amplified templated Ion Sphere™ Particles (ISPs) for sequencing on an Ion PGM™ Chip to obtain the essential data coverage. Sequencing was performed on an Ion PGM™ sequencer at Genotypic Technology’s Genomics facility, in Bengaluru, India.

### Quality control for reads and analysis

The samples were sequenced with Ion PGM Sequencer and analyzed with Torrent suite v 3.6. Once the base-calling was done, the raw reads underwent the in-built process of trimming and filtering to obtain only high-quality reads. Trimming was performed to evacuate any undesired base calls, adapter sequences, and lower-quality reads at the 3′end of the reads. Filtering at the next step removed reads judged to contain low-quality base calls. These two steps ensured the reads taken were of high quality; the clean reads were used for the subsequent analyses. The raw reads obtained were aligned to the reference *Gallus gallus* mitochondria (NC_001323.1) with the TMAP algorithm. The variants were detected using the inbuilt plugin Variant caller (V4.0) of TS.

### Validation of mtDNA genes with gene-specific primers

The mtDNA gene-specific primers were designed using IDT oligo analyzer software (Table 3). The mtDNA genes were amplified using the Prima-96™ Thermal Cycler (HIMEDIA). PCR amplification was performed in a 25 μL volume containing 1 μL of DNA template, 2.5 μL of 10 × PCR buffer with Mg^2+^, 2.5 μL of dNTP mixture (2.5 mM each), 1.25 μL of each primer (10 μM), 0.5 μL of Taq DNA polymerase (5 U/μL), and 16 μL of nuclease-free water. The PCR conditions were as follows: 95°C for 10 min; followed by 35 cycles of 94°C for 30 s, 53/58°C for 30 s, and 72°C for 45 s; and a final extension at 72°C for 10 min. The PCR products were detected on 2% agarose gel electrophoresis.

**TABLE 3:** The PCR primers for segmental amplification of chicken mtDNA genes.

S. No.	Gene name	mtDNA position	Primer sequences (5′-3′)	Tm (^o^C)	Amplification size (bp)
1	D-loop	270-336	F: GAATGGTTACCGGACATAAAC	50	67
			R: CCATTCATAGTTAGGTGACTT	49	
2	ND4	11366-11530	F: CCTTGTACCTATTCTAATACTG	47.5	165
			R: CATACTCTTGCCCACAGCC	55.9	
3	D-loop	216-436	F: CTCATTTACCCTCCCCATAA	50	221
			R: GTTGCTGATCTCTCGTGAG	51	
4	COX1	8050-8341	F: TATTCATCGTCTGAGAAGCCT	53.1	292
			R: TGGTTGGCCATGTGAGATG	55.3	
5	ND2	6008-6177	F: ATTAACCGGCTTCATGCCAG	55.7	170
			R: TTATGTGGTTTGATGAGTTGG	50.7	
6	rRNA	1996-2157	F: AGGTCAAGGTATAGCCTATGA	50	162
			R: GTATGTACGTGCCTCAGAG	51	
7	ND2	5529-5853	F: ATATTAACAATAGCAATCGCAAC	49.7	325
			R: AGGTGAGAATAGTGAGTTGTG	51.8	
8	ND4	12476-12796	F: AACACAAACTACGAACGGAT	48	321
			R: TATGAGAAGATGTTCTCGAG	48	
9	rRNA	3916-4253	F: TCAACTGCCAAGAACCCCC	57.8	338
			R: GATTGGCTCTTTAATGAATAGT	48.3	
10	CYTB	15115-15391	F: CAATACGGCTGACTCATCCA	52	277
			R: CTCAGGCTCACTCTACTAG	51	
11	D-loop	1150-1623	F: CACTTAACTCCCCTCACAAG	52	474
			R: TAGCTGGTGCAGATAACATG	50	
12	ND5	14683-15016	F: CCTCAATCCTCCTACATACT	50.4	333
			R: ACAGACTGCTAATAGGGAGC	53.8	
13	CYTB	14996-15477	F: GCTCCCTATTAGCAGTCTGT	52	482
			R: GATAGTAATACCTGCGATTGC	50	
14	D-loop	369-470	F: ACCCATTTGGTTATGCTCGC	52	101
			R: TGAGACTGGTCATGAAGTAC	50	
15	tRNA	6512-6625	F: CCCGGCACACTTTAGTGTG	53	114
			R: TGAAGCGTTAGGCTGTAGTC	52	

### Phylogenetic and molecular evolution analysis

To investigate the evolutionary relationships, a phylogenetic examination was performed using the total mitochondrial DNA and *D-loop* region sequences of seven native Indian chicken breeds along with twenty-two native Asian chicken breeds. Each of the sequence datasets was aligned by Clustal X and analyzed by neighbor-joining (N-J) in MEGA 10.0, and bootstrap analysis was performed with 1000 replications (Buehler and Baker, 2003; Thompson et al., 1997; Kumar et al., 2004). For building a neighbor-joining phylogenetic tree, Kimura’s two-parameters model was used for calculating the genetic distance of the haplotypes.

### Haplotypes diversity

Network analysis was used for haplotype diversity illustrated using NETWORK 10.1 (Bandelt et al., 1999). In chickens, population indices include several segregation sites (*S*), number of haplotypes (*H*), haplotype diversity (Hd), and nucleotide diversity (π), determined by the mtDNA *D-loop* sequences’ diversity and elucidated by the sequence polymorphism and the content of genetic variability (Nei, 1987). The DnaSP software version 10.1 was used for analysis and the alignment gaps arising from a deletion event were excluded from the calculations (Rozas et al., 2003). Between two sequences, the average number of nucleotide differences per site, known as nucleotide diversity (π), was defined as π = *n*/(*n* −1) Σ_ij_ *x*_*i*_ *x*_*j*_ *x*_*ij*_ or π = Σπ_*ij*_/*nc* [*n* = number of DNA sequences examined; *x*_*i*_ and *x*_*j*_ = frequencies of the *i*th and *j*th type of DNA sequences; π_*ij*_ = proportion of nucleotides in the respective types of DNA sequences; and *nc* = total number of sequence comparisons] (Nei, 1987). According to the Nei formula, the average heterozygosity or haplotype diversity, ℎ, is defined [ℎ = 2*n* (1—Σ*xi*2)/(2*n*—1); *xi* = frequency of haplotype and *n* = sample size] (Nei, 1987). The gene or haplotype frequencies were used for assessing the level of genetic differentiation among the population. The *F*st and *N*st significant tests by Arlequin software version 2.000 were used to explore the population’s genetic structure (Wright, 1951; Excoffier et al., 1992; Schneider et al., 2000).

## Results

The proposed whole mtDNA sequence analysis was effectively used to characterize the seven indigenous Indian chickens along with twenty-two native Asian chickens. The blood samples were collected and mtDNA was extracted from seven Indian native chicken breeds: *Aseel*, *Ghagus*, *Nicobari Brown*, *Tellichery*, *Kadaknath*, *Haringhata Black*, and *Red Jungle Fowl*. We successfully amplified mtDNA using an amplification method and NGS sequencing was done using an Ion PGM™ sequencer at Genotypic Technology’s Genomics facility. We obtained 757073, 89859, 105503, 50617, 15764, 264309, and 36685 sequencing reads from seven Indian native chickens, respectively (Table 4; Supplementary Table S1). The assembled complete mitochondrial genomes for seven Indian native chickens were submitted to Genbank under accession numbers, KP211418.1, KP211419.1, KP211422.1, KP211424.1, KP211425.1, KP211420.1, and KP211423.1 for *Aseel*, *Ghagus*, *Nicobari Brown*, *Tellichery*, *Kadaknath*, *Haringhata Black*, and *Indian Red Jungle Fowl*, respectively.

**TABLE 4:** Sequencing reads obtained from seven Indian native breeds.

	Aseel	Ghagus	Nicobari brown	Tellicherry	Kadaknath	Haringhata black	Indian Red Jungle fowl
Total number of reads	757073	89859	105503	50617	15764	264309	36685
Number of mapped reads	734474	87336	103692	49407	15224	257435	35693
Average base coverage depth	6303	593	799	387.1	90.22	1672	278.3
Uniformity of base coverage (%)	84.15	76.81	81.78	61.88	68.34	88.98	71.18
Genome base coverage at 1x (%)	100.00	100	100	100	99.68	100	100
Genome base coverage at 20x (%)	100.00	99.87	99.57	91.16	65.31	100	91.12

The complete length of mtDNA of Indian local chickens was 16,775 bp. Like most vertebrates, it contains a common structure, including 2 *rRNA* genes, 22 *tRNA* genes, 12 protein-coding genes, and 1 *D-loop* region (Lin et al., 2016; Gu and Li, 2020). The four nucleotides’ (i.e., A, T, G, and C) overall composition of assessed mtDNA was 30.26%, 23.76%, 32.48%, and 13.50%, in the order G > A > T > C, respectively. The inception codons for all the coding proteins was ATG, aside from *COX1* which is GTG (Table 5). The heavy (H) strand of mtDNA encoded all the mtDNA genes and the light (L) strand encoded four sorts of *tRNA* genes and *ND6* genes. Every one of these genes had 15 spaces in the length of 2–227 bp and had 3 overlaps in the length of 1-8 bp. These genes had three sorts of termination codons, namely TAA, TAG, and TGA. The “T– – “ is the 5′terminal of the adjoining gene (Anderson et al., 1981). The lengths of the two *rRNA* genes were 976 bp and 1621 bp. Among 12 protein-coding genes, the longest one was the *ND5* gene (1818 bp) and the most limited one was the *ND4L* gene (297 bp). As seen in other types of chicken, four *tRNA* genes were circulated in protein-coding genes, varying from 66 to 135 bp in size (Yu et al., 2016; Liu et al., 2016a; Liu et al., 2016b; Lin et al., 2019). The *D-loop* region was situated among *ND6* and *rRNA* with a length of 1227 bp (Table 5; Supplementary Table S2).

**TABLE 5:** Organization of the mitochondrial genome of seven native Indian chickens.

Gene name	Position		Size	Codon		Anticodon	Strand	Space/Overlap+
	Start	End		Start	Stop			
D-loop	1	1227	1227					
rRNA	1297	2272	976				H	70
rRNA	2346	3966	1621				H	74
ND1	4050	5024	975	ATG	TAA		H	84
ND2	5241	6281	1041	ATG	TAG		H	217
tRNA-Cys	6508	6573	66			GCA	L	227
COX1	6645	8132	1488	ATG	T--		H	72
tRNA-Ser	8124	8258	135			UGA/GCU	L	−8
tRNA-Asp	8261	8329	69			GUC	L	3
COX2	8331	9014	684	ATG	TAA		H	2
ATP6	9240	9923	884				H	226
COX3	9923	10706	784	ATG	T--		H	−1
ND3	10776	11126	351	ATG	TAA		H	70
ND4L	11196	11492	297	ATG	TAA		H	70
ND4	11486	12863	1378	ATG	T--		H	−6
ND5	13071	14888	1818	ATG	TAA		H	208
CYTB	14893	16035	1143	ATG	TAA		H	5
tRNA-Pro	16108	16177	70			UGG	L	73
ND6	16184	16705	522	ATG	TAA		L	7

T–−means incomplete termination codon; Negative numbers indicate overlapping nucleotides.

### Pattern of mtDNA *D-Loop* variability

**FIGURE 2:**
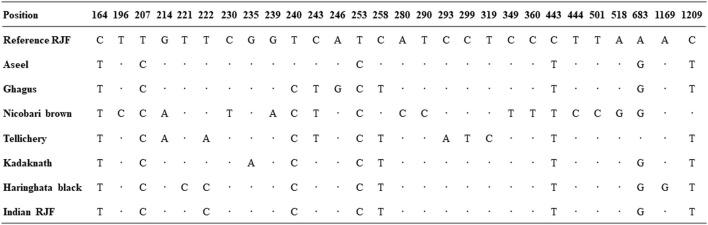
Pattern of mtDNA *D-loop* variability. Nucleotide polymorphisms were observed in the *D-loop* region of seven native Indian chicken sequences. Vertically oriented numbers indicate the site position and the sequences shown are only the variable sites. Dots (.) indicate identity with the reference sequence and different base letters denote substitution.

The 1227 bp mtDNA *D-loop* fluctuation design uncovered large variations between nucleotides 235 and 1209. In seven local Indian chicken breeds, eight haplotypes were related to variation at 28 destinations, and 8.33% of them were polymorphic (Figure 2). The total arrangement uncovered exceptionally high changeability in the mtDNA *D-loop* region between 164–360 bases; this variation comprises 23.5% of the seven successions. This rate is incredibly high contrasted with the other local chicken varieties at 5.54%–7.05% (Niu et al., 2002; Liu et al., 2004). This high pace of mtDNA *D-loop* variation might be credited to the relocation of birds all through the nation and diverse topographical districts in India. The base composition of the native Indian chicken breeds mtDNA *D-loop* shows that A+T grouping content was 60.39% while G+C was 39.61% (Ruokonen and Kvist, 2002).

### Sequence variation and haplotype distribution

Multiple sequence alignment was performed for seven native Indian chicken varieties and recognized eight haplotypes. The alignment of *D-loop* sequences was finished with a *gallus* reference sequence (NC_001323.1) utilizing Clustal-X. The accompanying domains and motifs were examined at the 5′end of the D-loop. Two units of invariant tetradecamer 5′- AACTATGAATGGTT-3′ were distinguished at positions 264 to 277 and 325 to 338. An interfered with thymine string (TTTTATTTTTTAA) was observed as conserved in all the individuals contemplated. There was likewise an intrusion of poly-C sequence (5′- CCCCCCCTTTCCCC-3′), which is generally conserved, and downstream to this there was a preserved sequence known as poly-G (5′-AGGGGGGGT-3′). Two moderated 5′- TACAT-3′ and 5′- TATAT-3′ were likewise found in all individuals. There were nine TATAT motifs and four TACAT found inside the *D-loop* and were, thus, conserved. The initial 163 base sets adjoining *tRNA* Glu were seen as exceptionally conserved in all individuals. The nucleotide replacements found in the nine variable haplotypes contained one G/T and two C/A transversions and the rest were all changes of which four were A/G transitions and seven were C/T transitions. This exhibits a solid predisposition towards transition. The C/T substitutions are more normal than the A/G substitution (Table 6).

**TABLE 6:** Each entry is the probability of substitution (r) from one base (row) to another base (column). Substitution pattern and rates were estimated under the Tamura and Nei. (1993) model (Tamura and Nei, 1993). Rates of different transitional substitutions are shown in bold and those of transversionsal substitutions are shown in italics. Relative values of instantaneous r should be considered when evaluating them. For simplicity, sum of r values is made equal to 100, The nucleotide frequencies are A = 30.26%, T/U = 23.76%, C = 32.48%, and G = 13.50%. For estimating ML values, a tree topology was automatically computed. The maximum Log likelihood for this computation was −23504.716. This analysis involved 9 nucleotide sequences. Codon positions included were 1st + 2nd + 3rd + Noncoding. There were a total of 16775 positions in the final dataset. Evolutionary analyses were conducted in MEGA X (Kumar et al., 2018).

Maximum likelihood estimate of substitution matrix				
	A	T/U	C	G
A	-	1.28	1.75	13.47
T/U	1.63	-	26.33	0.73
C	1.63	19.26	-	0.73
G	30.18	1.28	1.75	-

### Genetic distance among seven native Indian chicken breeds

The genetic distances within and between the seven native Indian chicken breeds were analyzed using the DnaSP program 10.1 (Table 7). The genetic distance within the seven chicken breeds was 0.000655–16.173895. Indian *RJF* chickens had the most noteworthy within-breed genetic distance, while *Ghagus* chickens had the least. Between the breeds, the genetic distance estimates went from 0.000655 to 16.188613. The genetic distance between the breeds was most noteworthy for Indian *RJF* and *Tellicherry* chickens, while it was least for *Ghagus* and *Aseel* chickens. An incredible number of variants and SNPs were recognized in *Nicobari Brown* and a minimal number of variants and SNPs were distinguished in *Tellicherry* and *Kadaknath*, respectively. The most number of INDELs were distinguished in *Kadaknath* and the least number of INDELs were recognized in *Tellicherry* (Table 8).

**TABLE 7:** Genetic distance between each pair among the seven Indian native chicken breeds.

Breed	Aseel	Ghagus	Nicobari brown	Tellicherry	Kadaknath	Haringhata black	Indian Red Jungle fowl	Reference Red Jungle fowl
Aseel								
Ghagus	0.000655							
Nicobari brown	0.003525	0.003344						
Tellicherry	0.002028	0.001729	0.003045					
Kadaknath	0.001132	0.001073	0.003225	0.001730				
Haringhata black	0.001371	0.001192	0.003464	0.001789	0.001073			
Indian Red Jungle Fowl	16.173895	16.138058	16.175363	16.188613	16.182522	16.123728		
Reference Red Jungle Fowl	0.002267	0.002447	0.003165	0.002507	0.002088	0.002447	16.154633	

**TABLE 8:** The number of variants, SNPs and INDELs identified in seven Indian chicken mitochondrial genome.

Sample ID	Total number of variants	Number of SNPs	Number of INDELs
Aseel	43	43	9
Ghagus	56	46	10
Nicobari brown	61	52	9
Tellicherry	42	35	7
Kadaknath	46	21	25
Haringhata black	54	41	13
Indian Red Jungle Fowl	43	34	9
Total	345	272	82

### Phylogenetic analysis of the haplotypes

**FIGURE 3:**
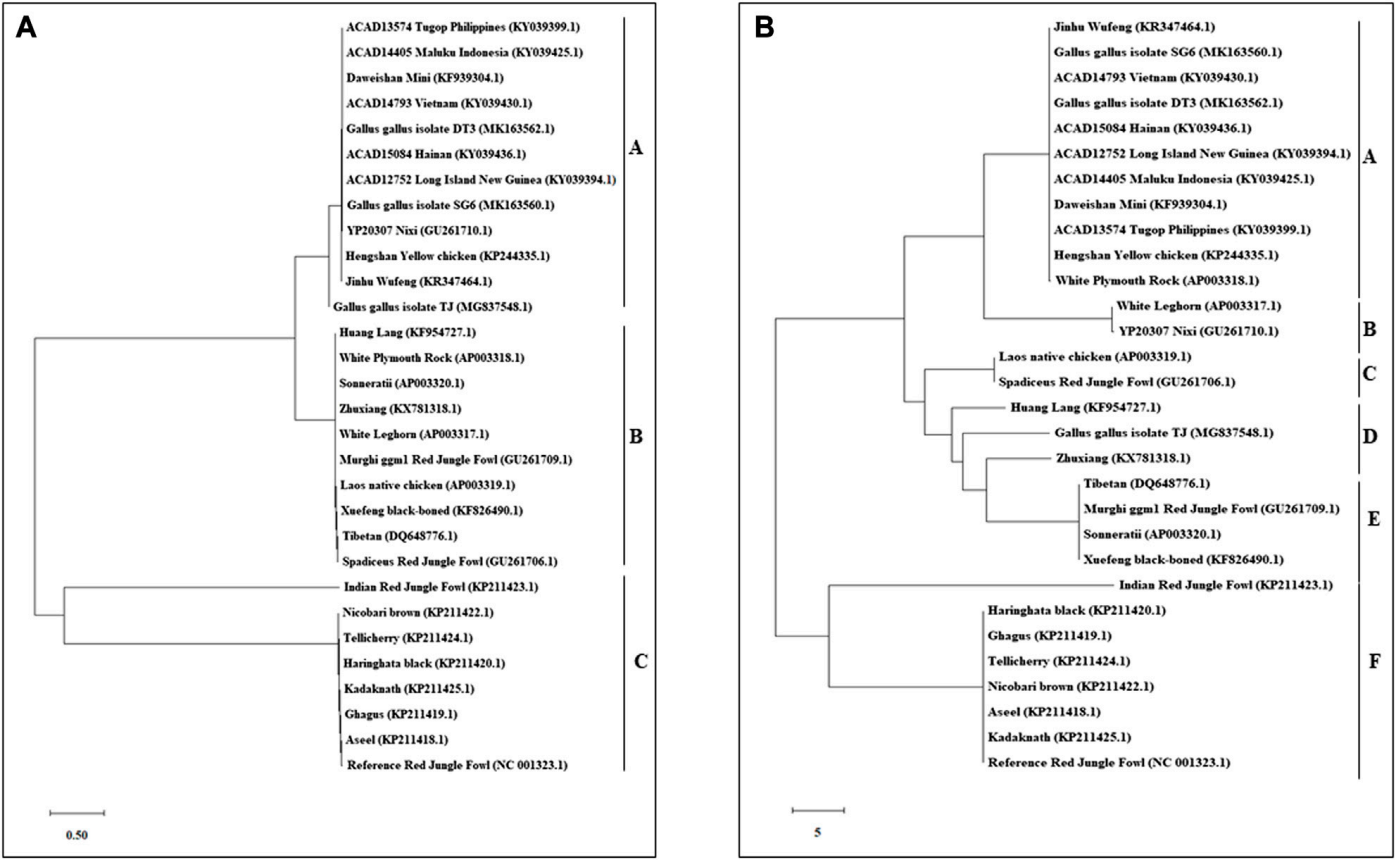
An N-J tree was constructed using MEGA 10.1 software. Phylogenetic analysis based on mtDNA *D-loop* region **(A)** and complete mtDNA genome sequences **(B)** of seven Indian native breads along with reference *RJF* mtDNA and twenty-two native Asian breeds sequences. The mtDNA genome sequences were obtained from the NGS sequencing and submitted in NCBI (Accession numbers: Aseel-KP211418.1; Ghagus-KP211419.1; Haringhata Black-KP211420.1; Kadaknath-KP211425.1; Nicobari Brown-KP211422.1; Indian Red Jungle Fowl-KP211423.1; Tellichery-KP211424.1). The numbers at the nodes represent the percentage bootstrap values for interior branches after 1000 replications.

An N-J tree indicated that the examined Asian native breeds and Indian local varieties were separated into six significant clades: A, B, C, D, E, and F. The native Indian breeds are situated at the base of the tree (Figure 3). This N-J tree produced from the total mitochondrial DNA of twenty-two native Asian breeds and seven local Indian varieties has comparative geographies. The clades A to E shared the native Asian breeds but clade F shared only native Indian breeds. Along these lines, our outcomes affirmed that *Kadaknath*-*H. black* and *Aseel*-*Ghagus* breeds have a nearby hereditary relationship. In addition, the N-J tree is generated from the *NADH dehydrogenase subunit* genes, *cytochrome c oxidase subunit* genes, mitochondrial encoded *ATP synthase membrane subunit 6* gene, *cytochrome b* gene, and *ribosomal RNA* genes. The results showed a close hereditary relationship with *Aseel*-*Ghagus* and *Nicobari brown*-Reference *RJF* (Figures 5–8).

### Network analysis

**FIGURE 4:**
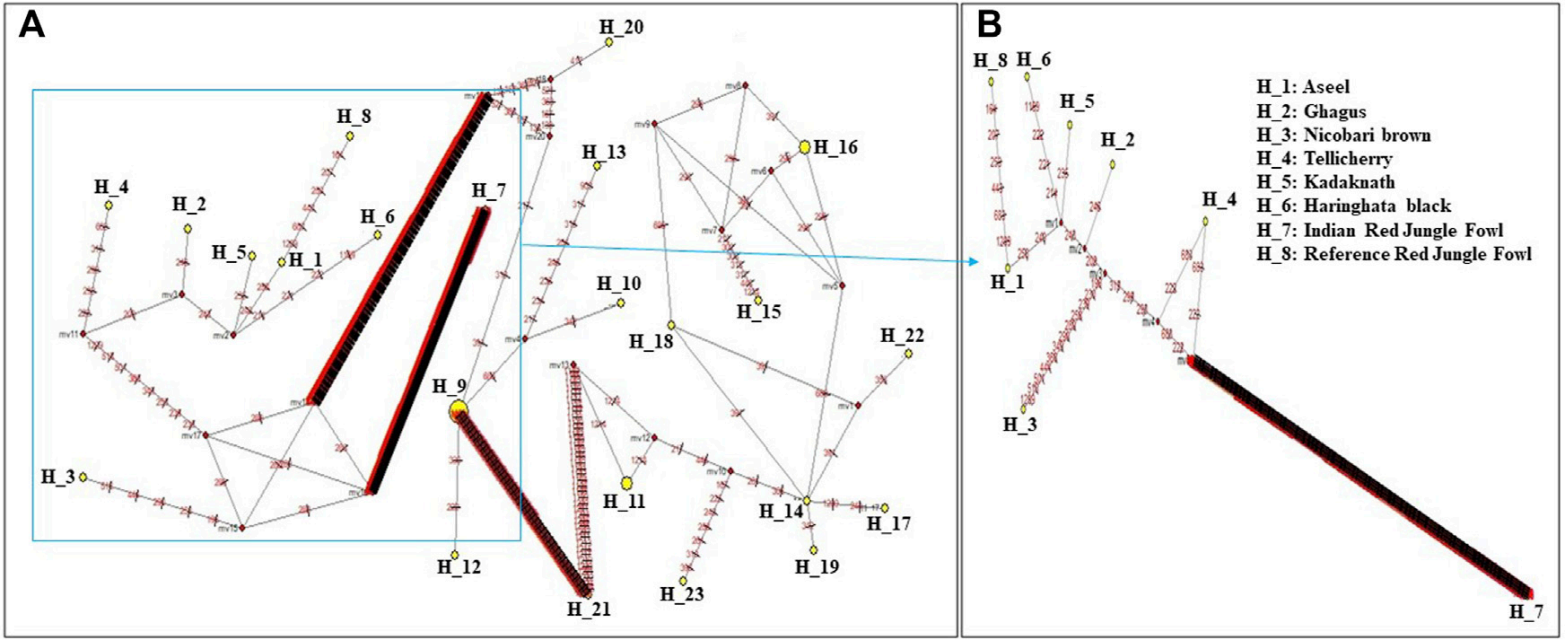
A median-joining network was produced by the network software 10.1 for eight haplotypes of native Indian chicken breeds along with reference mtDNA based on the polymorphic site of the mtDNA *D-loop* region **(A)** and complete mtDNA sequences **(B)**. The area of each yellow circle is proportional to the frequency of the corresponding haplotype. The pink dots illustrate median vectors (mv) and the numbers on each link line represent mutated positions.

**FIGURE 5:**
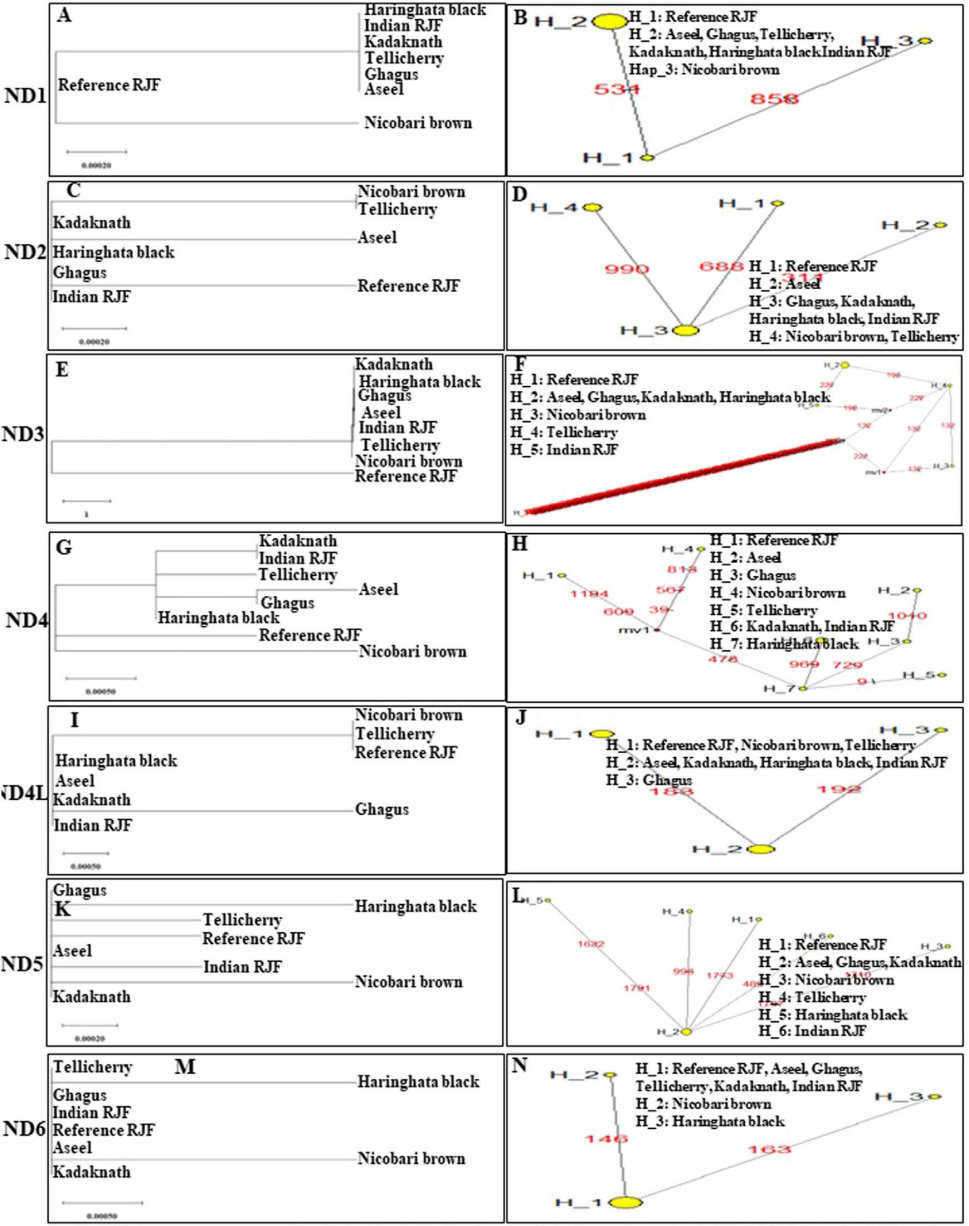
An N-J tree was constructed using MEGA 10.1 software. Phylogenetic analysis **(A, C, E, G, I, K, and M)** based on mtDNA *NADH dehydrogenase* subunit gene sequences of seven native Indian breeds along with reference *RJF*. The numbers at the nodes represent the percentage of bootstrap values for interior branches after 1,000 replications. A median-joining network was produced by the network software 10.1 for Indian native chicken breeds along with reference mtDNA based on polymorphic site of the mtDNA *NADH dehydrogenase* genes **(B, D, F, H, J, L, and N)**. The area of each yellow circle is proportional to the frequency of the corresponding haplotype. The pink dots illustrate median vectors (mv) and the numbers on each link line represent mutated positions.

**FIGURE 6:**
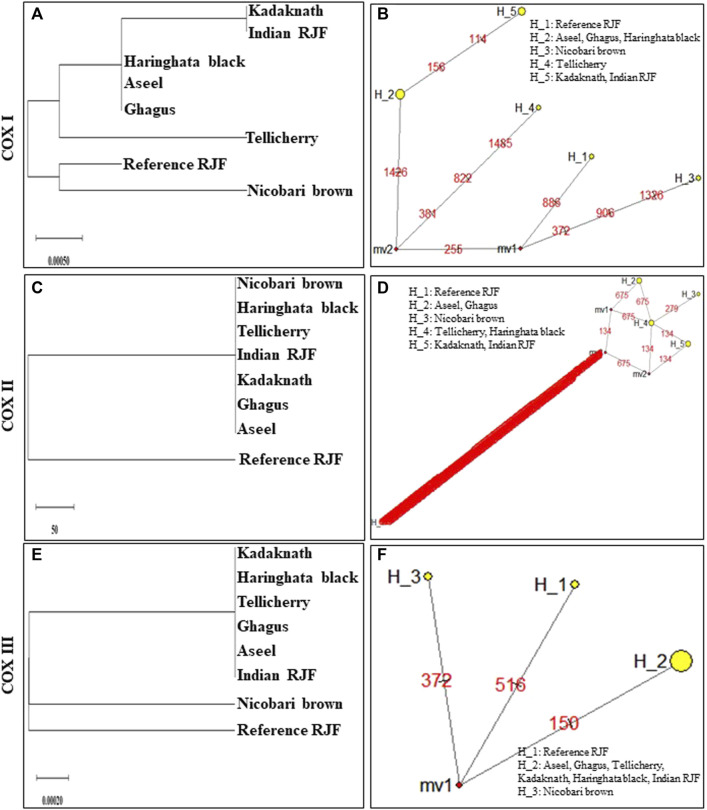
An N-J tree was constructed using MEGA 10.1 software. Phylogenetic analysis **(A,C,E)** based on mtDNA *Cytochrome c oxidase subunit* gene sequences of seven native Indian breeds along with reference *RJF*. The numbers at the nodes represent the percentage of bootstrap values for interior branches after 1000 replications. A median-joining network was produced by the network software 10.1 for native Indian chicken breeds along with reference mtDNA based on the polymorphic site of the mtDNA *Cytochrome c oxidase subunit* genes **(B,D,F)**. The area of each yellow circle is proportional to the frequency of the corresponding haplotype. The pink dots illustrate median vectors (mv) and the numbers on each link line represent mutated positions.

**FIGURE 7:**
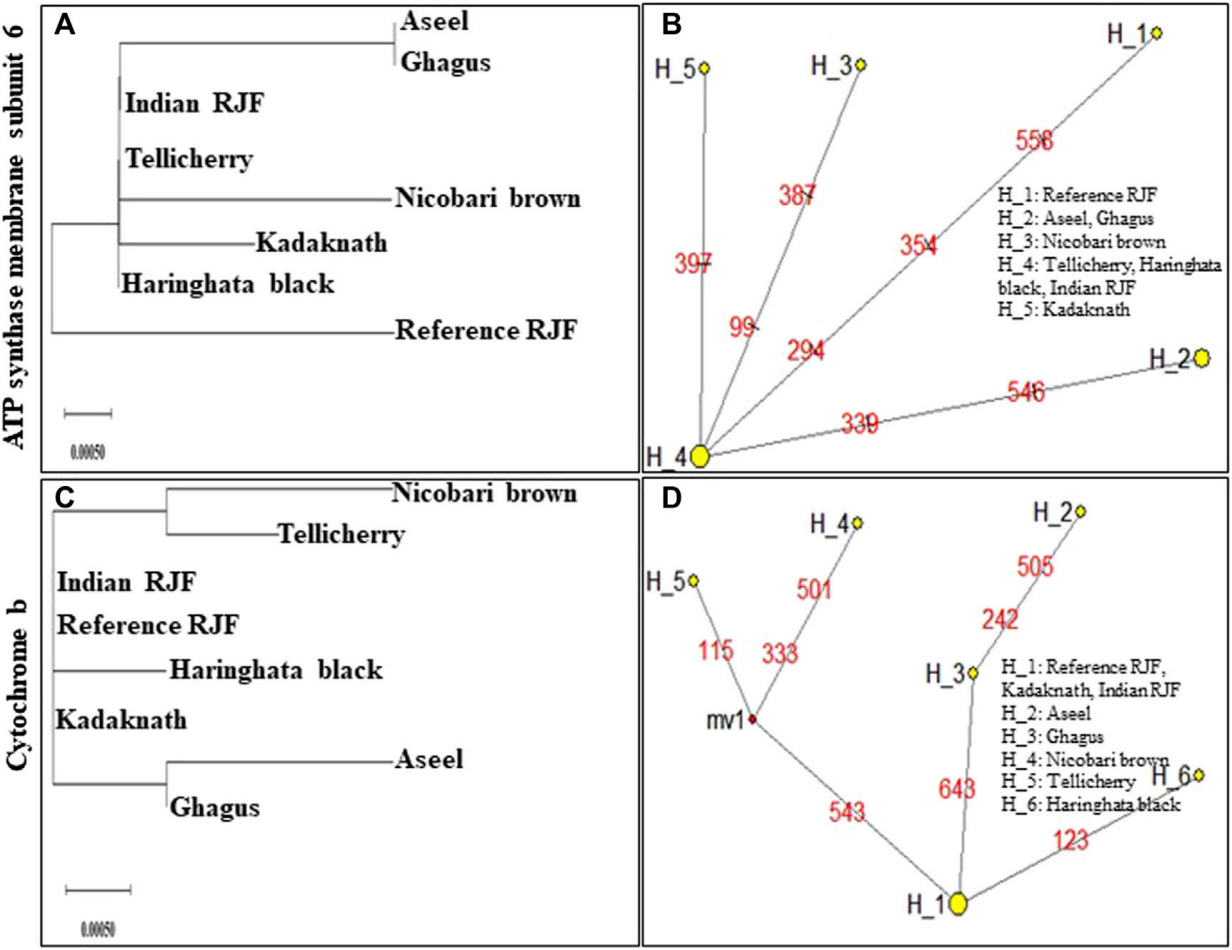
An N-J tree was constructed using MEGA 10.1 software. Phylogenetic analysis **(A,C)** based on mtDNA *ATP synthase membrane subunit 6* and *Cytochrome b* gene sequences of seven native Indian breeds along with reference *RJF*. The numbers at the nodes represent the percentage of bootstrap values for interior branch after 1000 replications. A median-joining network was produced by the network software 10.1 for native Indian chicken breeds along with reference mtDNA based on the polymorphic site of the mtDNA *ATP synthase membrane subunit 6* and *Cytochrome b* genes **(B,D)**. The area of each yellow circle is proportional to the frequency of the corresponding haplotype. The pink dots illustrate median vectors (mv) and the numbers on each link line represent mutated positions.

**FIGURE 8:**
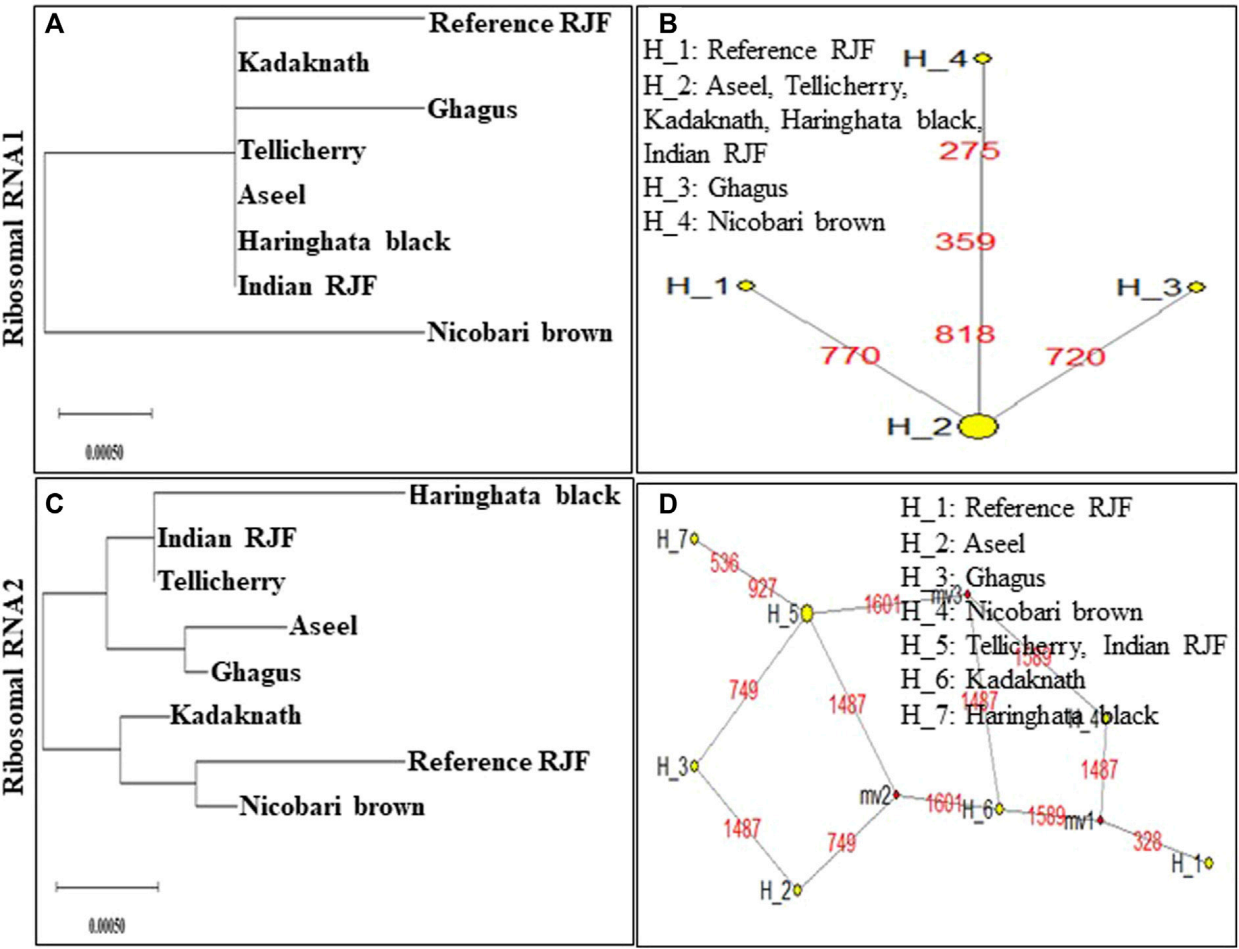
An N-J tree was constructed using MEGA 10.1 software. Phylogenetic analysis **(A,C)** based on mtDNA *Ribosomal RNA* 1 and 2 gene sequences of seven native Indian breeds along with reference *RJF*. The numbers at the nodes represent the percentage of bootstrap values for interior branches after 1000 replications. A median-joining network was produced by the network software 10.1 for native Indian chicken breeds along with reference mtDNA based on the polymorphic site of the mtDNA *Ribosomal RNA* 1 and 2 genes **(B,D)**. The area of each yellow circle is proportional to the frequency of the corresponding haplotype. The pink dots illustrate median vectors (mv) and the numbers on each link line represent mutated positions.

Median-joining networks were drawn for the eight haplotypes identified from the seven Indian native chickens along with reference *RJF* mtDNA, based on the variable characters of the complete alignment using the computer program NETWORK 10.1 (Bandelt et al., 1999). The results showed that the mtDNA sequence of Indian Red Jungle Fowl has the highest frequencies and this haplotype is connected to the frequencies of other haplotypes, forming star-like connections. It was also observed that there are mutational links to eight haplotypes with five median vectors (mv)∗ separating clades (Figure 4). The median-joining network examination was completed with the haplotypes from the native Indian breeds. The outcomes show that, out of the seven recognized native breeds haplotypes, just two breeds haplotypes i.e. *Kadaknath* and *Haringhata* black, showed uniqueness and fell into a different clade separated from other breeds. Also, median-joining networks were drawn for mtDNA structural genes, such as *NADH dehydrogenase subunit* genes, *cytochrome c oxidase subunit* genes, mitochondrial encoded *ATP synthase membrane subunit 6* gene, *cytochrome b* gene, and *ribosomal RNA* genes. The results showed that three breeds of haplotype i.e. *Indian RJF*, *Kadaknath*, and *H. black*, have uniqueness (Figures 5–8).

### The mtDNA genes validation

Genomic DNA was extracted from blood samples of seven native Indian chicken breeds and mtDNA genes were validated by PCR using gene-specific primers. PCR was able to amplify the segmental mtDNA region from all seven native breeds. Each expected PCR-amplified product demonstrated the sign band of approximately 67–482 bp in length on 2% agarose gel (Table 3; Supplementary Figures S2, S3). The Aseel-specific *ND4* primers (12476–12796 region) amplified all the breeds except *Ghagus*, whereas *Aseel rRNA*-specific primers (3916–4253 region) amplified all the breeds except *Ghagus* and *H. black*. The *Ghagus*-specific *ND4* primers (11366–11530 region) amplified *Indian RJF*, *Nicobari*, *Kadaknath*, and *Ghagus* but did not amplify *Aseel*, *H. black*, and *Tellicherry*, whereas *Ghagus rRNA*-specific primers (1996–2157 region) amplified all the breeds except *Tellicherry*. The *Kadaknath COX1* specific primers (8050–8341 region) amplified all the breeds except *Tellicherry*.

## Discussion

It is hypothesized that chickens (*Gallus gallus domesticus*) were domesticated from wild jungle fowls in Southeast Asia approximately 10,000 years ago (Ahmed et al., 2017). Native fowl extinction can be improved by modifying cultivation, taking care of the chickens, and better prosperity spread. However, selection and crossbreeding can be used for genetic improvement. Aside from conventional breeding, molecular breeding techniques (Microsatellite markers and SNPs), functional genomics, gene silencing, and genome editing techniques can be used to improve the quality of traits in chickens. Microsatellite markers, SSCP, and sequencing techniques can be used to distinguish proof of polymorphism on genes and assessment of the impact of polymorphism on growth traits in chickens. Molecular genetics has given a few useful assets to exploit the extraordinary abundance of polymorphism at the DNA level, for example, DNA-based genetic markers. The advantage of mtDNA is that it is present in higher copy numbers within the cells and thus can be recovered even from highly degraded specimens (Fumihito et al., 1996). For species identification, forensic studies, and anthropological and evolutionary research, the mitochondrial genome SNPs are assuming a significant role (Yacoub and Fathi, 2013). In 2016, single nucleotide polymorphism was observed, and eleven SNPs in the *D-loop* region of chicken mitochondrial DNA were reported (Jia et al., 2016).

To set up a phylogenetic relationship, studies were conducted on domestic fowl, different RJF subspecies, and other jungle fowls. In 1994, an investigation proposed that *G.g. gallus* is a major or sole contributor to a single domestication event, and three further species of *RJF* mtDNA *D-loop* region sequences were taken for phylogenetic analysis. This proved *G.g. gallus* is the real matriarchic origin of all the domestic poultry (Fumihito et al., 1994; Fumihito et al., 1996). In 2009, two ribosomal genes (*12S rRNA* and *16S rRNA*) were used to study the genetic divergence between Indian RJF (*G.g. murghi*), Gallus gallus subspecies (*G.g. spadicus*, *G.g. gallus,* and *G.g. bankiva*) including *G.g. domesticus* (domestic fowl), and three other Gallus species (*G. varius*, *G. lafayetei*, and *G. sonneratii*) and found that, compared to other jungle fowls, *G.g. murghi* was closer to *Gallus gallus* subspecies, however between Indian *RJF* and other *RJF* subspecies, the divergence was very low (Gupta et al., 2009). Furthermore, the study found no noticeable contrasts between *G.g. spadicus* and *G.g. gallus* yet *G.g. bankiva* showed contrasts with both the genetic and phylogenetic relationships among these species (Ulfah et al., 2016). Recent studies confirmed multiple domestications of Indian and other domestic chickens and provided evidence for the domestication of Indian birds from *G.g. spadiceus*, *G.g. gallus*, and *G.g. murghi* (Kanginakudru et al., 2008; Mekchay et al., 2014; Maw et al., 2015). These studies avoided the two different subspecies of red jungle fowl i.e. *G.g. murghi* and *G.g. jabouillei*, however, they provided a framework for genetic studies in wild jungle fowls and native and domestic chicken breeds. Considering solid confirmations of the domestication of chicken in the Indus valley, it might be interesting to contemplate the genetic relatedness between *Indian RJF* and other *RJF* subspecies including *G.g. domesticus* and other jungle fowls (Kawabe et al., 2014).

Indian *RJFs* are widely distributed across 51 × 10^5^ km^2^ in 21 states of India (Fernandes et al., 2009; Mogilicherla et al., 2022) (Figure 1). We were more interested to explore the phylogenetic relationship of Indian *RJF* with reference *RJF* and native Asian breeds along with other native Indian breeds and understand the contribution of Indian *RJF*, *G. g. murghi*, to the domestication event. Hence, in the present study, seven native Indian breeds (*Aseel*, *Ghagus*, *Nicobari brown*, *Kadaknath*, *Tellicherry*, *H. black*, and *Indian Red Jungle fowl*) and twenty-two native Asian breeds were used to study the nucleotide sequence variation in complete mtDNA and the most variable region of mtDNA *D-loop* region to build up the phylogenetic relationship among the Indian breeds (Table 1; Table 2). Currently, no sequence data is accessible from these seven native Indian breeds. However, one of these breeds is likely to have been a contributor to one of the earliest known chicken domestication events, for example in the Mohanjo-Daro Indus valley.

The preserved traits like Struthioniformes, Falconiformes, and Sphenisciformes have been depicted across a variety of avian species (Haring et al., 2001; Wani et al., 2014). The Indian indigenous chicken *D-loop* area sequence has cytosines and guanine strings close to each other, making the advancement of a consistent hairpin structure possible (Quinn and Wilson, 1993). In all neighborhood chickens of India, the conserved sequence motifs of TACAT and TATAT were found. Such subjects are depicted as termination-associated sequence segments (TASs) with mtDNA synthesis (Foran et al., 1988). Both *Galliformes* and mammals have TASs, which may propose a strong auxiliary capacity of the *D-loop* area of the two genera, while the lack of variety in TASs among the *Galliformes* may be a direct result of the particular utilitarian constraints. The eight haplotypes recognized from the Indian indigenous chicken and the phylogenetic examination spoke to the formative associations.

A large number of the investigations on domestic chicken origins have focused on the *D-Loop*; there were additionally a few examinations concentrating on mitochondrial genomics to analyze domestic chicken origins (Miao et al., 2013). Kauai feral chicken mitochondrial phylogenies (*D-loop* and whole mtDNA) revealed two different clades within their samples (Gering et al., 2015). Maybe the reason for the difference was that the samples for the examination were different between the whole mtDNA phylogeny and mtDNA *D-loop* phylogeny. We found that the mitochondrial phylogenies (*D-loop* and whole Mt genome) uncovered a few clades, and their examination re-evaluated the worldwide mtDNA profiles of chickens and encouraged our study of the settlement in India.

From the mtDNA examination, we saw that Indian *RJF* is the origin of reference *RJF* as well as Indian breeds (i.e. *Aseel*, *Ghagus*, *Nicobari brown*, *Kadaknath*, *H. black*, and *Tellicherry*). The reference *RJF* is closely related to the Indian reference *RJF*, which, in turn, has contributed to the genetic makeup of six native Indian breeds. This is likewise true for the Indian chicken, which originated from independent domestication from Indian *RJF* and likely from other *RJF* species. Curiously, the mixing of various Indian breeds with Indian *RJF* could explain how the current-day Asiatic chicken may have originated from various ancestors through numerous domestication events and such multi-origin breeds could still be seen in a single geographical location. This is in agreement with the current-day perceptions of native Japanese chickens that originated from various regions (Oka et al., 2007). However, all the breeds aside from Indian *RJF* form a solitary group, suggesting a common ancestor for these birds, including jungle fowls and domestic birds. The separation of the Indian *RJF* from the main group of breeds shows the possibility of a speciation event. Our examination uncovered that the Indian breeds are relatively pure with uncommon hybridization with reference *RJF*. In the current investigation of Indian breeds, we did not come across recognizable hybridization at least in the recent past, as shown by a clear separation of Indian *RJF* clades from reference *RJF* in mtDNA-based phylogeny. All these results indicate the genetic integrity of the Indian *RJF*.

In high-altitude birds, hypoxia is an unavoidable environmental stress; in natural selection, every step of aerobic respiration had to be experienced to improve the adaptability to hypoxia (Altshuler and Dudley, 2006; Scott, 2011; Scott and Milsom, 2006). Mitochondria play an important role in aerobic respiration through oxidative phosphorylation, because the majority of produced cell ATP is consumed for cellular oxygen uptake (Chandel and Schumacker, 2000; Xu et al., 2007). To determine the role of mitochondrial genes in high-altitude adaptation, six high-altitude Phasianidae birds and 16 low-altitude relatives’ mitochondrial genomes were analyzed, and four lineages were found for this high-altitude habitat (Zhou et al., 2014). Their results strongly suggest that the adaptive evolution of mitochondrial genes, i.e. *ND2*, *ND4*, and *ATP6,* played a critical role during the independent acclimatization to high altitude by galliform birds. In Tibet, a chicken breed was found to have a missense mutation in the *MT-ND5* subunit of the *NADH dehydrogenase* gene for high-altitude adaptation (Bao et al., 2007). To explore the regulatory mechanisms for hypoxia adaptability, the *ATP-6* gene was sequenced from 28 Tibetan chickens and 29 Chinese domestic chickens; six SNPs were detected (Zhao et al., 2015). In high-altitude adaption, *cytochrome c oxidase* (*COX*) was the key mitochondrial gene and plays an important role in oxidative phosphorylation regulation and oxygen sensing transfer. For identifying the *COX* gene SNP, the Tibet Chicken and four lowland chicken breeds (Dongxiang Chicken, Silky Chicken, Hubbard ISA White broiler, and Leghorn layer) were used and 13 haplotypes were defined for the 14 SNPs. It was concluded that the significant difference in *MT-CO3* gene mutation might have a relationship with the high-altitude adaptation (Bao et al., 2008). *MT-CO3* gene was sequenced by using 125 Tibetan chickens and 144 Chinese domestic chickens; eight SNPs were identified, and were defined into nine haplotypes. They found positive and negative haplotype associations with high-altitude adaptation (Sun et al., 2013). However, the *MT-COI* gene was sequenced from 29 Tibetan chickens and 30 Chinese domestic chickens, and nine SNPs were detected (Zhao et al., 2015). In our study, *ND3*, *ND4*, *ND5*, *ATP6*, *COX1*, and *COX2* showed high-altitude lineages between *Nicobari brown* and Reference *RJF* birds which may help chickens to evolve to adapt to Nicobari Island environments (Figures 5–8).

mtDNA mutations contribute to enclosing both tissue-specific and multiple-system disorders in human diseases (Taylor and Turnbull, 2005; Tuppen et al., 2010). The spindle-associated chromosomal exchange did not show antagonistic consequences for fertilization on subsequent embryo/fetal development in the rhesus monkey (Tachibana et al., 2009). Subsequently, this method may speak to another dependable restorative way to deal with the transmission of mtDNA mutations in influenced families. Mitochondrial heterogeneity is the presence of at least two kinds of mitochondrial (mt) DNA in the same individual/tissue/cell and it is firmly related to animal health and disease. In mtDNA, *ND2* is a protein-coding gene and it partakes in the mitochondrial respiratory chain and oxidative phosphorylation. In cloned sequencing of the *ND2* region, numerous potential heteroplasmic locales were recognized, which possibly reflected bountiful heteroplasmy in the chicken mitochondrial genome (Yang et al., 2020). These outcomes give a significant reference for further research on heteroplasmy in chicken mitochondria. Recently, an examination utilized complete mtDNA from *tuberculosis* patients’ blood samples and explored the conceivable mtDNA variations (Tonsing et al., 2020). Twenty-eight non-synonymous variants were found and most of the variations lie in the *D-loop* of the non-protein-coding region of the mitochondrial DNA. Runting and stunting syndrome (RSS) generally happens early in life and causes low body weight and extensive economic losses in the commercial broiler industry (Kang et al., 2012). In sex-linked dwarf (SLD) chickens, the RSS is related to mitochondria dysfunction, and mutations in the TWNK gene are one reason for mtDNA exhaustion (Li et al., 2019; Hu et al., 2020). We recommend that mutations in the mitochondrial genome should be validated further to fully understand their relationship with animal diseases.

## Limitations of the work

Deep sampling using the NGS technique offers a straightforward, high-throughput, and cost-effective platform for effectively detecting and measuring mitochondrial heteroplasmy in complete mitochondrial genomes. The main limitation of this research is that only seven native Indian breeds were used. Nevertheless, the information that was collected will be useful in finding heteroplasmy in various chicken breeds both domestically and internationally.

## Conclusion

In summary, the eight haplotypes of the native chicken population showed a relatively rich genetic pool, and molecular information on genetic diversity revealed may help in developing genetic improvement and conservation strategies to better utilize precious genetic reserves. The phylogenetic relationship of native Indian chicken breeds using entire mtDNA and D-loop sequences showed that the South East Asian *RJF* is distant from the Indian *RJF* but genetically close to the Indian breeds, whereas the Indian *Aseel* breed was more closely related to the reference *RJF* than the Indian *RJF*. The grouping of Indian *RJF* separated from Indian native chickens and the presence of subcontinent explicit haplogroups gives additional proof for an independent domestication event of chickens in the subcontinent. For high-altitude hypoxic adaptation, it is important to improve the efficiency of oxygen usage instead of enhancing oxygen uptake and transport. Thus, in natural selection, the mitochondrial genome encoded 12 essential structural genes (6 *NADH dehydrogenase* genes, *cytochrome b subunit*, 3 *cytochrome c oxidase*, and *ATP synthase subunit*), which must have mutated during adaptation to high-altitude hypoxic conditions.

## Data Availability

The datasets presented in this study can be found in online repositories. The names of the repository/repositories and accession number(s) can be found in the article/Supplementary Material.

## Supplementary material

SUPPLEMENTARY FIGURE S1 PCR products are loaded on 1% agarose gel. Samples A1-A11-*Aseel*; A13-A23-*Ghagus*; B1-B11-*Nicobari brown*; B13-B23-*Nicobari black*; C1-C11-*Tellichery*; C13-C23-*Kadaknath*; D1-D11-*Haringhata*; D13-D23-*Red Jungle Fowl*; A12, B12, C12, D12 are ladder.

SUPPLEMENTARY FIGURE S2 Validation of mtDNA *D-loop* **(A)** and *NADH dehydrogenase* gene **(B)** regions with *D-loop* and *NADH* gene-specific primers, respectively. F-Female; M-Male; 1-100 bp DNA ladder; 2-negative control; 3- Indian *RJF*; 4- *Nicobari*; 5- *Kadaknath*; 6- *Ghagus*; 7- *Aseel*; 8- *Haringhata black*; 9- *Tellicherry*.

SUPPLEMENTARY FIGURE S3 Validation of mtDNA *COX1*, *tRNA*, *rRNA*, and *CYTB* gene regions with gene-specific primers, respectively. F-Female; M-Male; 1-100 bp DNA ladder; 2-negative control; 3- Indian *RJF*; 4- *Nicobari*; 5- *Kadaknath*; 6- *Ghagus*; 7- *Aseel*; 8- *Haringhata black*; 9- *Tellicherry*.

SUPPLEMENTARY TABLE S1 Single nucleotide variants (SNVs) called in whole mtDNA sequencing analysis of samples from seven native Indian chicken breeds. The results from sequencing 500ng total mtDNA extracted from whole blood.

SUPPLEMENTARY TABLE S2 Organization of the mitochondrial genome in seven native Indian chicken breeds.

## Abbreviations

*ATP6*, mitochondrial encoded ATP synthase membrane subunit 6; *COX1*, cytochrome c oxidase subunit I; *COX2*, cytochrome c oxidase subunit II; *COX3*, cytochrome c oxidase subunit III; *CYTB*, Cytochrome b; INDELs, Insertions, and deletions; ICAR-NBAGR, Indian council for the agricultural research-National bureau of animal genetic resources; mtDNA, Mitochondrial DNA, NGS, Next generation sequencing; *NAD1*, NADH dehydrogenase subunit 1; *NAD2*, NADH dehydrogenase subunit 2; *NAD3*, NADH dehydrogenase subunit 3; *NAD4*, NADH dehydrogenase subunit 4; *NAD5*, NADH dehydrogenase subunit 5; *NAD6*, NADH dehydrogenase subunit 6; OXPHOS, oxidative phosphorylation; RJF, Red Jungle Fowl; rRNA, Ribosomal RNA; RSS, Runting and stunting syndrome; SNP, Single-nucleotide polymorphism; SEA, South East Asia; SLD, sex-linked dwarf; TWNK, Twinkle mtDNA helicase.
