# Supplementary figures and images for: Indian Red Jungle fowl reveals a genetic relationship with South East Asian Red Jungle fowl and Indian native chicken breeds as evidenced through whole mitochondrial genome sequences
Source: Front Genet. 2023 Aug 9;14:1083976. doi: 10.3389/fgene.2023.1083976 (PMC10445952; doi:10.3389/fgene.2023.1083976)

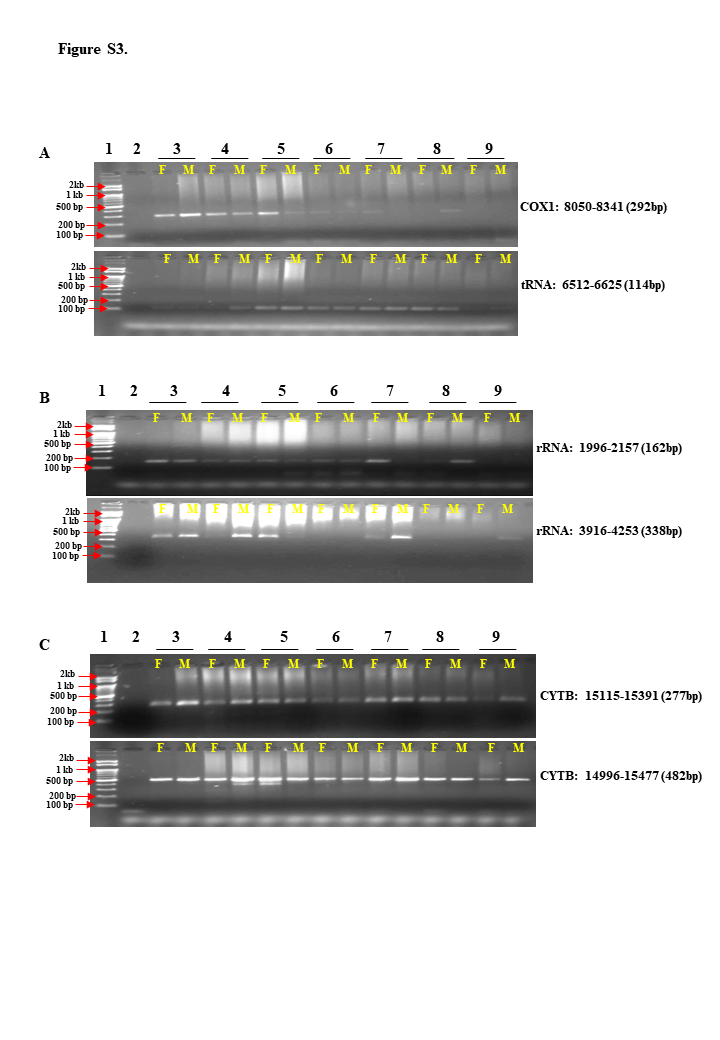

Supplement: Supplementary file 1 [file Image3.tif]

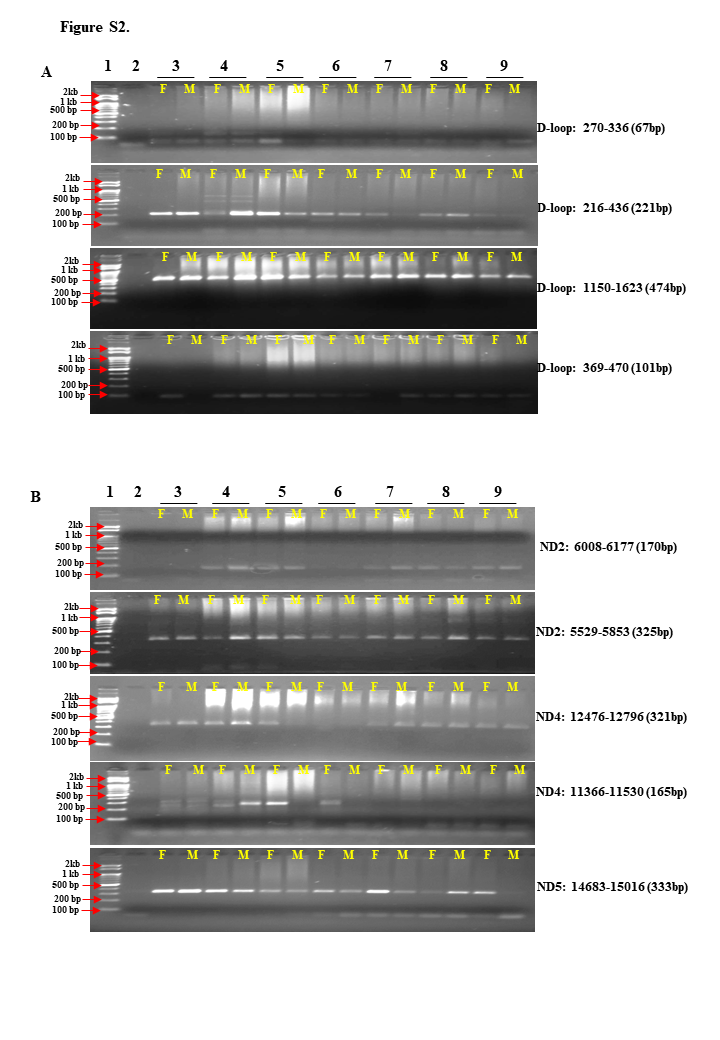

Supplement: Supplementary file 2 [file Image2.tif]

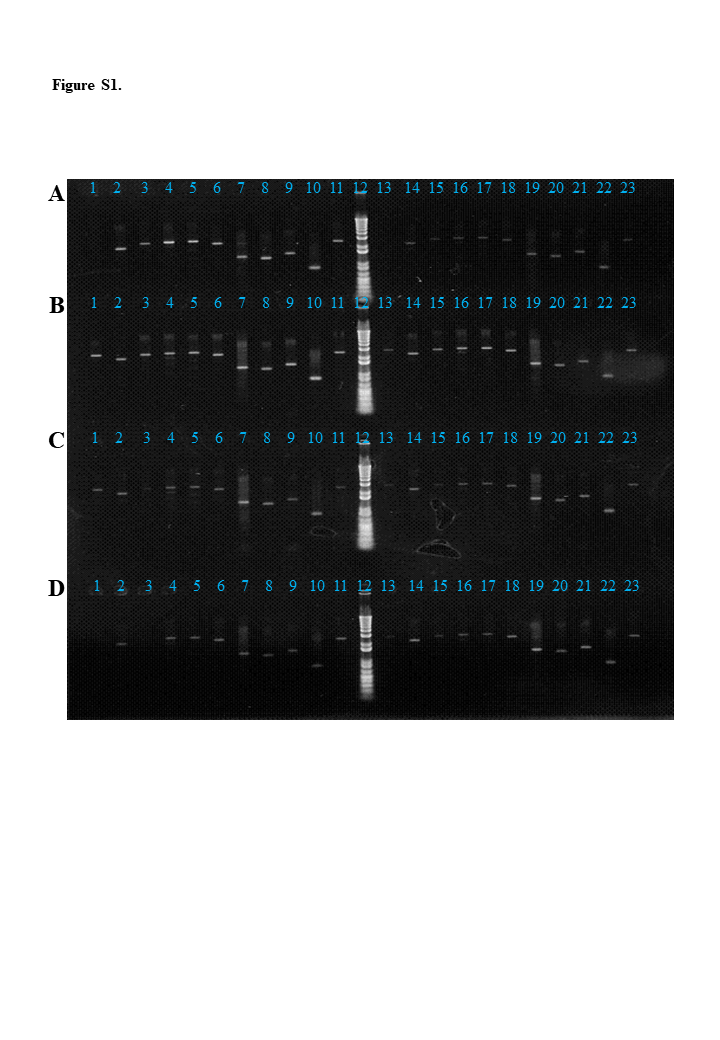

Supplement: Supplementary file 3 [file Image1.tif]
