# Supplementary material for: Indian Red Jungle fowl reveals a genetic relationship with South East Asian Red Jungle fowl and Indian native chicken breeds as evidenced through whole mitochondrial genome sequences
Source: Front Genet. 2023 Aug 9;14:1083976. doi: 10.3389/fgene.2023.1083976 (PMC10445952; doi:10.3389/fgene.2023.1083976)
Supplement: Supplementary file 4 [file Table1.doc]

| **S. No.** | **Position** | **Aseel** | | | **Ghagus** | | | **Nicobari brown** | | | **Tellicherry** | | | **Kadaknath** | | | **Haringhata black** | | | **Red Jungle Fowl** | | |
| --- | --- | --- | --- | --- | --- | --- | --- | --- | --- | --- | --- | --- | --- | --- | --- | --- | --- | --- | --- | --- | --- | --- |
|  |  | **RA** | **AA** | **HE/HO** | **RA** | **AA** | **HE/HO** | **RA** | **AA** | **HE/HO** | **RA** | **AA** | **HE/HO** | **RA** | **AA** | **HE/HO** | **RA** | **AA** | **HE/HO** | **RA** | **AA** | **HE/HO** |
| 1 | 235 |  |  |  |  |  |  |  |  |  |  |  |  | G | A | HO |  |  |  |  |  |  |
| 2 | 239 |  |  |  |  |  |  | G | A | HO |  |  |  |  |  |  |  |  |  |  |  |  |
| 3 | 240 | T | C | HO | T | C | HO | T | C | HO | T | C | HO | T | C | HO | T | C | HO | T | C | HO |
| 4 | 243 | C | T | HO | C | T | HO | C | T | HO | C | A,T | HO |  |  |  | C | T | HE |  |  |  |
| 5 | 246 | A | G | HE | A | G | HE |  |  |  |  |  |  |  |  |  |  |  |  |  |  |  |
| 6 | 253 | T | C | HO | T | C | HO | T | C | HO | T | C | HO | T | C | HO | T | C | HO | T | C | HO |
| 7 | 258 | C | T | HO | C | T | HO |  |  |  | C | T | HO | C | T | HO | C | T | HO | C | T | HO |
| 8 | 280 |  |  |  |  |  |  | A | C | HO |  |  |  |  |  |  |  |  |  |  |  |  |
| 9 | 290 |  |  |  |  |  |  | T | C | HO |  |  |  |  |  |  |  |  |  |  |  |  |
| 10 | 293 |  |  |  |  |  |  |  |  |  | C | A | HO |  |  |  |  |  |  |  |  |  |
| 11 | 299 |  |  |  |  |  |  |  |  |  | C | T | HO |  |  |  |  |  |  |  |  |  |
| 12 | 307 | C | T | HE | C | T | HE | C | T | HE |  |  |  | C | T | HE | C | T | HE | C | T | HE |
| 13 | 308 | C | T | HE | C | T | HE | C | T | HE |  |  |  |  |  |  |  |  |  |  |  |  |
| 14 | 319 |  |  |  |  |  |  |  |  |  | T | C | HO |  |  |  |  |  |  |  |  |  |
| 15 | 349 |  |  |  |  |  |  | C | T | HO |  |  |  |  |  |  |  |  |  |  |  |  |
| 16 | 352 | T | C | HE | T | C | HE |  |  |  |  |  |  |  |  |  |  |  |  |  |  |  |
| 17 | 360 |  |  |  |  |  |  | C | T | HO |  |  |  |  |  |  |  |  |  |  |  |  |
| 18 | 388 |  |  |  |  |  |  |  |  |  | C | T | HE |  |  |  |  |  |  |  |  |  |
| 19 | 443 | C | T | HO | C | T | HE |  |  |  | C | T | HO | C | T | HO | C | T | HO | C | T | HO |
| 20 | 501 |  |  |  |  |  |  | T | C | HO |  |  |  |  |  |  |  |  |  |  |  |  |
| 21 | 518 |  |  |  |  |  |  | A | G | HO |  |  |  |  |  |  |  |  |  |  |  |  |
| 22 | 683 | A | G | HO | A | G | HO | A | G | HO |  |  |  | A | G | HO | A | G | HO | A | G | HO |
| 23 | 960 |  |  |  | G | T | HE |  |  |  |  |  |  |  |  |  |  |  |  |  |  |  |
| 24 | 971 |  |  |  | A | T | HE |  |  |  |  |  |  |  |  |  | A | T | HE |  |  |  |
| 25 | 982 |  |  |  | A | T | HE | A | T | HE |  |  |  |  |  |  | A | T | HE |  |  |  |
| 26 | 1002 | A | T | HE |  |  |  | A | T | HE |  |  |  |  |  |  | A | T | HE |  |  |  |
| 27 | 1169 |  |  |  |  |  |  |  |  |  |  |  |  |  |  |  | A | G | HO |  |  |  |
| 28 | 1209 | C | T | HO | C | T | HO |  |  |  | C | T | HO |  |  |  | C | T | HO |  |  |  |
| 29 | 1571 |  |  |  |  |  |  | A | G | HO |  |  |  |  |  |  |  |  |  |  |  |  |
| 30 | 1655 |  |  |  |  |  |  | T | C | HO |  |  |  |  |  |  |  |  |  |  |  |  |
| 31 | 2016 |  |  |  | G | A | HE |  |  |  |  |  |  |  |  |  |  |  |  |  |  |  |
| 32 | 2066 | T | C | HO | T | C | HO | T | C | HO | T | C | HO | T | C | HO | T | C | HO | T | C | HO |
| 33 | 2114 |  |  |  |  |  |  | A | G | HO |  |  |  |  |  |  |  |  |  |  |  |  |
| 34 | 2115 |  |  |  |  |  |  | C | G | HE |  |  |  |  |  |  |  |  |  |  |  |  |
| 35 | 2401 |  |  |  | C | A | HE |  |  |  |  |  |  |  |  |  |  |  |  |  |  |  |
| 36 | 2673 | C | T | HO | C | T | HO | C | T | HO | C | T | HO | C | T | HO | C | T | HO | C | T | HO |
| 37 | 2881 |  |  |  |  |  |  |  |  |  |  |  |  |  |  |  | T | C | HO |  |  |  |
| 38 | 3094 | C | T | HE | C | T | HO |  |  |  |  |  |  |  |  |  |  |  |  |  |  |  |
| 39 | 3272 |  |  |  |  |  |  |  |  |  |  |  |  |  |  |  | C | A | HO |  |  |  |
| 40 | 3832 | G | A,C | HO | G | A | HO | G | A | HO | G | C | HO | G | A | HE | G | A | HO | G | A | HO |
| 41 | 3934 | T | C | HO |  |  |  |  |  |  |  |  |  |  |  |  |  |  |  |  |  |  |
| 42 | 4580 | G | A | HO | G | A | HO |  |  |  | G | A | HE |  |  |  | G | A | HO | G | A | HO |
| 43 | 4907 |  |  |  |  |  |  | G | A | HE |  |  |  |  |  |  |  |  |  |  |  |  |
| 44 | 5551 | T | C | HO |  |  |  |  |  |  |  |  |  |  |  |  |  |  |  |  |  |  |
| 45 | 5928 | C | A | HO | C | A | HE | C | A | HE |  |  |  |  |  |  | C | A | HO |  |  |  |
| 46 | 6027 |  |  |  | A | G | HE |  |  |  |  |  |  |  |  |  |  |  |  |  |  |  |
| 47 | 6530 |  |  |  |  |  |  |  |  |  | A | G | HO |  |  |  |  |  |  |  |  |  |
| 48 | 6758 |  |  |  |  |  |  |  |  |  |  |  |  | T | C | HE |  |  |  | T | C | HO |
| 49 | 6800 |  |  |  |  |  |  |  |  |  |  |  |  | T | C | HO |  |  |  | T | C | HO |
| 50 | 6819 |  |  |  | G | A | HE |  |  |  |  |  |  |  |  |  |  |  |  |  |  |  |
| 51 | 6899 | A | G | HO | A | G | HO |  |  |  | A | G | HO | A | G | HO | A | G | HO | A | G | HO |
| 52 | 7016 |  |  |  |  |  |  | C | T | HO |  |  |  |  |  |  |  |  |  |  |  |  |
| 53 | 7025 |  |  |  |  |  |  |  |  |  | G | A | HO |  |  |  |  |  |  |  |  |  |
| 54 | 7466 |  |  |  |  |  |  |  |  |  | C | T | HO |  |  |  |  |  |  |  |  |  |
| 55 | 7530 | C | G | HO | C | G | HO | C | G | HE | C | G | HE | C | G | HO | C | G | HO | C | G | HO |
| 56 | 7550 |  |  |  |  |  |  | A | G | HO |  |  |  |  |  |  |  |  |  |  |  |  |
| 57 | 7970 |  |  |  |  |  |  | T | C | HO |  |  |  |  |  |  |  |  |  |  |  |  |
| 58 | 8070 | T | C | HO | T | C | HO |  |  |  |  |  |  |  |  |  | T | A,C | HE | T | C | HE |
| 59 | 8129 |  |  |  |  |  |  |  |  |  | T | C | HE |  |  |  |  |  |  |  |  |  |
| 60 | 8183 |  |  |  |  |  |  | G | A | HE |  |  |  |  |  |  |  |  |  |  |  |  |
| 61 | 8330 | T | C | HO | T | C | HO |  |  |  |  |  |  |  |  |  | T | C | HO | T | C | HO |
| 62 | 8464 |  |  |  |  |  |  |  |  |  |  |  |  | T | C | HE |  |  |  | T | C | HO |
| 63 | 8609 | T | C | HO | T | C | HO |  |  |  |  |  |  |  |  |  | T | C | HO | T | C | HO |
| 64 | 8787 | A | G | HE | A | G | HE |  |  |  |  |  |  |  |  |  |  |  |  |  |  |  |
| 65 | 9005 | G | A | HO | G | A | HO |  |  |  |  |  |  |  |  |  |  |  |  |  |  |  |
| 66 | 9338 |  |  |  |  |  |  | A | G | HO |  |  |  |  |  |  |  |  |  |  |  |  |
| 67 | 9533 | A | G | HO | A | G | HE | A | G | HO |  |  |  |  |  |  | A | G | HO | A | G | HO |
| 68 | 9578 | C | T | HO | C | T | HO |  |  |  |  |  |  |  |  |  |  |  |  |  |  |  |
| 69 | 9593 | G | A | HO | G | A | HO | G | A | HO |  |  |  |  |  |  | G | A | HO | G | A | HO |
| 70 | 9626 |  |  |  |  |  |  | A | G | HO |  |  |  |  |  |  |  |  |  |  |  |  |
| 71 | 9785 | A | T | HO | A | T | HO |  |  |  |  |  |  |  |  |  |  |  |  |  |  |  |
| 72 | 9797 | G | A | HO | G | A | HO | G | A | HO | G | A | HE | G | A | HO | G | A | HO | G | A | HO |
| 73 | 10072 | A | G | HO | A | G | HO |  |  |  | A | G | HO | A | G | HO | A | G | HO | A | G | HO |
| 74 | 10294 |  |  |  |  |  |  | A | G | HE |  |  |  |  |  |  |  |  |  |  |  |  |
| 75 | 10438 | T | C | HO | T | C | HO | T | C | HO | T | C | HO | T | C | HE | T | C | HO | T | C | HO |
| 76 | 10907 |  |  |  |  |  |  | A | T | HO |  |  |  |  |  |  |  |  |  |  |  |  |
| 77 | 10968 | T | C | HO | T | C | HO |  |  |  |  |  |  |  |  |  | T | C | HE | T | C | HO |
| 78 | 10997 | T | C | HE | T | C | HE | T | C | HE |  |  |  |  |  |  | T | C | HE | T | C | HE |
| 79 | 11378 | C | T | HO | C | T | HO |  |  |  |  |  |  | C | T | HO | C | T | HO | C | T | HO |
| 80 | 11387 |  |  |  | A | G | HO |  |  |  |  |  |  |  |  |  |  |  |  |  |  |  |
| 81 | 11494 |  |  |  |  |  |  |  |  |  | G | A | HE |  |  |  |  |  |  |  |  |  |
| 82 | 11524 |  |  |  |  |  |  | C | T | HE |  |  |  |  |  |  |  |  |  |  |  |  |
| 83 | 11950 | T | C | HE | T | C | HE | T | C | HE |  |  |  |  |  |  |  |  |  | T | C | HE |
| 84 | 11963 | C | T | HO | C | T | HO |  |  |  | C | T | HO | C | T | HE | C | T | HE | C | T | HO |
| 85 | 12052 |  |  |  |  |  |  | C | T | HO |  |  |  |  |  |  |  |  |  |  |  |  |
| 86 | 12094 | T | C | HO | T | C | HO | T | C | HO | T | C | HO | T | C | HE | T | C | HO | T | C | HO |
| 87 | 12098 |  |  |  |  |  |  |  |  |  |  |  |  |  |  |  |  |  |  | G | A | HE |
| 88 | 12214 | C | T | HO | C | T | HO |  |  |  |  |  |  |  |  |  |  |  |  |  |  |  |
| 89 | 12298 |  |  |  |  |  |  | A | G | HO |  |  |  |  |  |  |  |  |  |  |  |  |
| 90 | 12323 |  |  |  | A | T | HE |  |  |  |  |  |  | A | T | HE |  |  |  |  |  |  |
| 91 | 12419 |  |  |  |  |  |  |  |  |  |  |  |  |  |  |  |  |  |  |  |  |  |
| 92 | 12454 |  |  |  |  |  |  |  |  |  |  |  |  | T | C | HE |  |  |  | T | C | HE |
| 93 | 12495 | C | T | HO |  |  |  |  |  |  |  |  |  |  |  |  |  |  |  |  |  |  |
| 94 | 12679 | T | C | HO | T | C | HE | T | C | HO | T | C | HO | T | C | HE | T | C | HO |  |  |  |
| 95 | 13559 |  |  |  |  |  |  |  |  |  |  |  |  |  |  |  |  |  |  | T | C | HO |
| 96 | 13704 |  |  |  | C | A | HE |  |  |  |  |  |  | C | A | HE |  |  |  | C | A | HE |
| 97 | 14066 |  |  |  |  |  |  |  |  |  | T | C | HO |  |  |  |  |  |  |  |  |  |
| 98 | 14334 |  |  |  |  |  |  |  |  |  |  |  |  |  |  |  |  |  |  |  |  |  |
| 99 | 14694 |  |  |  | C | A | HE |  |  |  |  |  |  |  |  |  | C | A | HE |  |  |  |
| 100 | 14702 |  |  |  |  |  |  |  |  |  |  |  |  |  |  |  | C | T | HO |  |  |  |
| 101 | 14780 |  |  |  |  |  |  | T | C | HO |  |  |  |  |  |  |  |  |  |  |  |  |
| 102 | 14843 | C | T | HO | C | T | HO | C | T | HO | C | T | HE |  |  |  | C | T | HO |  |  |  |
| 103 | 14861 |  |  |  |  |  |  |  |  |  |  |  |  |  |  |  | T | C | HO |  |  |  |
| 104 | 14867 |  |  |  |  |  |  | G | A | HO |  |  |  |  |  |  |  |  |  |  |  |  |
| 105 | 15007 |  |  |  |  |  |  |  |  |  | G | A | HO |  |  |  |  |  |  |  |  |  |
| 106 | 15015 |  |  |  |  |  |  |  |  |  |  |  |  |  |  |  | C | T | HO |  |  |  |
| 107 | 15134 | G | A | HO |  |  |  |  |  |  |  |  |  |  |  |  |  |  |  |  |  |  |
| 108 | 15137 |  |  |  |  |  |  |  |  |  | A | G | HE |  |  |  |  |  |  |  |  |  |
| 109 | 15222 |  |  |  |  |  |  |  |  |  | C | T | HE |  |  |  |  |  |  |  |  |  |
| 110 | 15225 |  |  |  |  |  |  | G | A | HE |  |  |  |  |  |  |  |  |  |  |  |  |
| 111 | 15393 |  |  |  |  |  |  | G | A | HO |  |  |  |  |  |  |  |  |  |  |  |  |
| 112 | 15420 |  |  |  |  |  |  |  |  |  |  |  |  |  |  |  | T | C | HE |  |  |  |
| 113 | 15435 |  |  |  |  |  |  | T | C | HO | T | C | HO |  |  |  |  |  |  |  |  |  |
| 114 | 15535 | G | A | HO | G | A | HO |  |  |  |  |  |  |  |  |  |  |  |  |  |  |  |
| 115 | 15938 |  |  |  |  |  |  |  |  |  | T | G | HE |  |  |  |  |  |  | T | G | HE |
| 116 | 16121 |  |  |  |  |  |  | G | A | HO | G | A | HO |  |  |  |  |  |  |  |  |  |
| 117 | 16329 |  |  |  |  |  |  | G | A | HO |  |  |  |  |  |  |  |  |  |  |  |  |
