# Supplementary material for: Indian Red Jungle fowl reveals a genetic relationship with South East Asian Red Jungle fowl and Indian native chicken breeds as evidenced through whole mitochondrial genome sequences
Source: Front Genet. 2023 Aug 9;14:1083976. doi: 10.3389/fgene.2023.1083976 (PMC10445952; doi:10.3389/fgene.2023.1083976)
Supplement: Supplementary file 5 [file Table2.doc]

| **S. No.** | **Position** | **Gene** | **Reference**  **RJF** | **Aseel** | **Ghagus** | **Nicobari Brown** | **Tellicherry** | **Kadaknath** | **Haringhata Black** | **Indian RJF** |
| --- | --- | --- | --- | --- | --- | --- | --- | --- | --- | --- |
| 1 | 235 | **D-loop**  (1-1227) | G |  |  |  |  | A |  |  |
| 2 | 239 |  | G |  |  | A |  |  |  |  |
| 3 | 240 |  | T | C | C | C | C | C | C | C |
| 4 | 243 |  | C | T | T | T | A,T |  | T |  |
| 5 | 246 |  | A | G | G |  |  |  |  |  |
| 6 | 253 |  | T | C | C | C | C | C | C | C |
| 7 | 258 |  | C | T | T |  | T | T | T | T |
| 8 | 280 |  | A |  |  | C |  |  |  |  |
| 9 | 290 |  | T |  |  | C |  |  |  |  |
| 10 | 293 |  | C |  |  |  | A |  |  |  |
| 11 | 299 |  | C |  |  |  | T |  |  |  |
| 12 | 307 |  | C | T | T | T |  | T | T | T |
| 13 | 308 |  | C | T | T | T |  |  |  |  |
| 14 | 319 |  | T |  |  |  | C |  |  |  |
| 15 | 349 |  | C |  |  | T |  |  |  |  |
| 16 | 352 |  | T | C | C |  |  |  |  |  |
| 17 | 360 |  | C |  |  | T |  |  |  |  |
| 18 | 388 |  | C |  |  |  | T |  |  |  |
| 19 | 443 |  | C | T | T |  | T | T | T | T |
| 20 | 501 |  | T |  |  | C |  |  |  |  |
| 21 | 518 |  | A |  |  | G |  |  |  |  |
| 22 | 683 |  | A | G | G | G |  | G | G | G |
| 23 | 960 |  | G |  | T |  |  |  |  |  |
| 24 | 971 |  | A |  | T |  |  |  | T |  |
| 25 | 982 |  | A |  | T | T |  |  | T |  |
| 26 | 1002 |  | A | T |  | T |  |  | T |  |
| 27 | 1169 |  | A |  |  |  |  |  | G |  |
| 28 | 1209 |  | C | T | T |  | T |  | T |  |
| 29 | 1571 | **rRNA**  (1297-3966) | A |  |  | G |  |  |  |  |
| 30 | 1655 |  | T |  |  | C |  |  |  |  |
| 31 | 2016 |  | G |  | A |  |  |  |  |  |
| 32 | 2066 |  | T | C | C | C | C | C | C | C |
| 33 | 2114 |  | A |  |  | G |  |  |  |  |
| 34 | 2115 |  | C |  |  | G |  |  |  |  |
| 35 | 2401 |  | C |  | A |  |  |  |  |  |
| 36 | 2673 |  | C | T | T | T | T | T | T | T |
| 37 | 2881 |  | T |  |  |  |  |  | C |  |
| 38 | 3094 |  | C | T | T |  |  |  |  |  |
| 39 | 3272 |  | C |  |  |  |  |  | A |  |
| 40 | 3832 |  | G | A,C | A | A | C | A | A | A |
| 41 | 3934 |  | T | C |  |  |  |  |  |  |
| 42 | 4580 | **ND1**  (4050-5024) | G | A | A |  | A |  | A | A |
| 43 | 4907 |  | G |  |  | A |  |  |  |  |
| 44 | 5551 | **ND2**  (5241-6281) | T | C |  |  |  |  |  |  |
| 45 | 5928 |  | C | A | A | A |  |  | A |  |
| 46 | 6027 |  | A |  | G |  |  |  |  |  |
| 47 | 6530 | **tRNA-Cys**  (6508-6573) | A |  |  |  | G |  |  |  |
| 48 | 6758 | **COX1**  (6645-8132) | T |  |  |  |  | C |  | C |
| 49 | 6800 |  | T |  |  |  |  | C |  | C |
| 50 | 6819 |  | G |  | A |  |  |  |  |  |
| 51 | 6899 |  | A | G | G |  | G | G | G | G |
| 52 | 7016 |  | C |  |  | T |  |  |  |  |
| 53 | 7025 |  | G |  |  |  | A |  |  |  |
| 54 | 7466 |  | C |  |  |  | T |  |  |  |
| 55 | 7530 |  | C | G | G | G | G | G | G | G |
| 56 | 7550 |  | A |  |  | G |  |  |  |  |
| 57 | 7970 |  | T |  |  | C |  |  |  |  |
| 58 | 8070 |  | T | C | C |  |  |  | A,C | C |
| 59 | 8129 | **tRNA-Ser**  (8124-8258) | T |  |  |  | C |  |  |  |
| 60 | 8183 |  | G |  |  | A |  |  |  |  |
| 61 | 8330 | **tRNA-Asp**  (8261-8329) | T | C | C |  |  |  | C | C |
| 62 | 8464 | **COX2**  (8331-9014) | T |  |  |  |  | C |  | C |
| 63 | 8609 |  | T | C | C |  |  |  | C | C |
| 64 | 8787 |  | A | G | G |  |  |  |  |  |
| 65 | 9005 |  | G | A | A |  |  |  |  |  |
| 66 | 9338 | **ATP6**  (9240-9923) | A |  |  | G |  |  |  |  |
| 67 | 9533 |  | A | G | G | G |  |  | G | G |
| 68 | 9578 |  | C | T | T |  |  |  |  |  |
| 69 | 9593 |  | G | A | A | A |  |  | A | A |
| 70 | 9626 |  | A |  |  | G |  |  |  |  |
| 71 | 9785 |  | A | T | T |  |  |  |  |  |
| 72 | 9797 |  | G | A | A | A | A | A | A | A |
| 73 | 10072 | **COX3**  (9923-10706) | A | G | G |  | G | G | G | G |
| 74 | 10294 |  | A |  |  | G |  |  |  |  |
| 75 | 10438 |  | T | C | C | C | C | C | C | C |
| 76 | 10907 | **ND3**  (10776-11126) | A |  |  | T |  |  |  |  |
| 77 | 10968 |  | T | C | C |  |  |  | C | C |
| 78 | 10997 |  | T | C | C | C |  |  | C | C |
| 79 | 11378 | **ND4L**  (11196-11492) | C | T | T |  |  | T | T | T |
| 80 | 11387 |  | A |  | G |  |  |  |  |  |
| 81 | 11494 | **ND4**  (11486-12863) | G |  |  |  | A |  |  |  |
| 82 | 11524 |  | C |  |  | T |  |  |  |  |
| 83 | 11950 |  | T | C | C | C |  |  |  | C |
| 84 | 11963 |  | C | T | T |  | T | T | T | T |
| 85 | 12052 |  | C |  |  | T |  |  |  |  |
| 86 | 12094 |  | T | C | C | C | C | C | C | C |
| 87 | 12098 |  | G |  |  |  |  |  |  | A |
| 88 | 12214 |  | C | T | T |  |  |  |  |  |
| 89 | 12298 |  | A |  |  | G |  |  |  |  |
| 90 | 12323 |  | A |  | T |  |  | T |  |  |
| 91 | 12419 |  | G |  |  |  |  |  |  |  |
| 92 | 12454 |  | T |  |  |  |  | C |  | C |
| 93 | 12495 |  | C | T |  |  |  |  |  |  |
| 94 | 12679 |  | T | C | C | C | C | C | C |  |
| 95 | 13559 | **ND5**  (13071-14888) | T |  |  |  |  |  |  | C |
| 96 | 13704 |  | C |  | A |  |  | A |  | A |
| 97 | 14066 |  | C |  |  |  | C |  |  |  |
| 98 | 14334 |  | T |  |  |  |  |  |  |  |
| 99 | 14694 |  | C |  | A |  |  |  | A |  |
| 100 | 14702 |  | C |  |  |  |  |  | T |  |
| 101 | 14780 |  | C |  |  | C |  |  |  |  |
| 102 | 14843 |  | C | T | T | T | T |  | T |  |
| 103 | 14861 |  | T |  |  |  |  |  | C |  |
| 104 | 14867 |  | G |  |  | A |  |  |  |  |
| 105 | 15007 | **CYTB**  (14893-16035) | G |  |  |  | A |  |  |  |
| 106 | 15015 |  | C |  |  |  |  |  | T |  |
| 107 | 15134 |  | G | A |  |  |  |  |  |  |
| 108 | 15137 |  | A |  |  |  | G |  |  |  |
| 109 | 15222 |  | C |  |  |  | T |  |  |  |
| 110 | 15225 |  | G |  |  | A |  |  |  |  |
| 111 | 15393 |  | G |  |  | A |  |  |  |  |
| 112 | 15420 |  | T |  |  |  |  |  | C |  |
| 113 | 15435 |  | T |  |  | C | C |  |  |  |
| 114 | 15535 |  | G | A | A |  |  |  |  |  |
| 115 | 15938 |  | T |  |  |  | G |  |  | G |
| 116 | 16121 | **tRNA-Pro**  (16108-16177) | G |  |  | A | A |  |  |  |
| 117 | 16329 | **ND6**  (16184-16705) | G |  |  | A |  |  |  |  |
